# The role of inflammatory response and metabolic reprogramming in sepsis-associated acute kidney injury: mechanistic insights and therapeutic potential

**DOI:** 10.3389/fimmu.2024.1487576

**Published:** 2024-10-31

**Authors:** An-Bu Liu, Bin Tan, Ping Yang, Na Tian, Jin-Kui Li, Si-Cong Wang, Li-Shan Yang, Lei Ma, Jun-Fei Zhang

**Affiliations:** ^1^ Department of Emergency Medical, General Hospital of Ningxia Medical University, Yinchuan, Ningxia, China; ^2^ Ningxia Key Laboratory of Clinical and Pathogenic Microbiology, General Hospital of Ningxia Medical University, Yinchuan, China; ^3^ School of Clinical Medicine, Ningxia Medical University, Yinchuan, Ningxia, China; ^4^ Department of Emergency Medical, Yanchi County People’s Hospital, Wuzhong, Ningxia, China

**Keywords:** sepsis associated acute kidney injury (SA-AKI), inflammatory response, metabolic reprogramming, regulatory cell death (RCD), mechanism

## Abstract

Sepsis represents a severe condition characterized by organ dysfunction resulting from a dysregulated host response to infection. Among the organs affected, the kidneys are particularly vulnerable, with significant functional impairment that markedly elevates mortality rates. Previous researches have highlighted that both inflammatory response dysregulation and metabolic reprogramming are crucial in the onset and progression of sepsis associated acute kidney injury (SA-AKI), making these processes potential targets for innovative therapies. This study aims to elucidate the pathophysiological mechanisms of renal injury in sepsis by perspective of inflammatory response dysregulation, with particular emphasis on pyroptosis, necroptosis, autophagy, and ferroptosis. Furthermore, it will incorporate insights into metabolic reprogramming to provide a detailed analysis of the mechanisms driving SA-AKI and explore potential targeted therapeutic strategies, providing solid theoretical framework for the development of targeted therapies for SA-AKI.

## 1 Introduction

Sepsis is a potentially life-threatening condition characterized by organ dysfunction resulting from a dysregulated host response to infection (1). Its characteristics include severe systemic inflammation and multi-organ dysfunction resulting from a dysregulated host response to infection. Epidemiological studies indicate that the mortality rate among sepsis patients can reach up to 20.6% (2, 3). In cases of septic shock, the mortality rate can escalate to 40%-50% (4). Additionally, the management of sepsis can be complicated by multidrug-resistant pathogens, adverse drug reactions, and other negative events, which can subsequently lead to increased morbidity and mortality rates (5–7). Current management strategies for sepsis predominantly involve fluid resuscitation, infection control, vasopressor administration, and organ support therapies; however, their effectiveness remains suboptimal. Innovative approaches, including Extracorporeal Membrane Oxygenation (ECMO) and Remote Ischemic Conditioning (RIC), are emerging as potential adjuncts for cardiac and pulmonary support in sepsis patients. Nonetheless, the prohibitive cost of these technologies exacerbates the financial burden on patients, and their availability is generally restricted to large tertiary care centers, hindering broader implementation. Furthermore, the complex etiology of sepsis poses challenges for early diagnosis and treatment. Consequently, elucidating the underlying mechanisms of sepsis is vital for the development of targeted therapeutic strategies and for enhancing patient survival outcomes.

Sepsis initially manifests as a systemic inflammatory response syndrome (SIRS), which can be managed through interventions such as antimicrobial therapy. As the condition progresses, it may lead to dysfunction or failure of multiple organs, including the heart, brain, kidneys, and lungs, which is a principal factor contributing to the high mortality rate associated with sepsis. Sepsis-associated acute kidney injury (SA-AKI) is one of the most prevalent complications of sepsis and significantly elevates the mortality risk of affected patients (8). The primary clinical manifestation of sepsis-associated acute kidney injury (SA-AKI) is a sudden deterioration in renal function in septic patients. This is characterized by elevated blood urea nitrogen (BUN) and serum creatinine levels, alongside a decrease in the glomerular filtration rate (GFR) and urine output (9). Despite ongoing research, the pathogenesis of sepsis-associated acute kidney injury (SA-AKI) remains inadequately understood. Contributing factors include dysregulated inflammatory responses, hemodynamic disturbances, endothelial dysfunction in renal vasculature, and mitochondrial dysfunction (10). These areas of investigation are currently fragmented, necessitating a cohesive synthesis to propel future research. This review will systematically analyze the potential pathophysiological mechanisms of SA-AKI, with an emphasis on inflammatory dysregulation and metabolic reprogramming. It might also delineate the well-established renal impairment pathways associated with sepsis and offer a succinct overview of prevailing treatment strategies and prospective therapeutic targets.

## 2 Dysregulated inflammatory response in SA-AKI

The Systemic Inflammatory Response Syndrome (SIRS) plays a crucial role in the onset of sepsis and the subsequent development of organ dysfunction. In the context of sepsis-induced systemic immune responses, pathogen-associated molecular patterns (PAMPs) and damage-associated molecular patterns (DAMPs) effectively activate the innate immune response. This process typically involves the complement system, coagulation cascade, and activation of endothelial cells (11). The excessive inflammatory response driven by PAMPs and DAMPs can lead to impairment of endothelial barrier function in blood vessels and organs following regulated cell death (RCD). This ultimately manifests as dysregulation in the release of pro-inflammatory and anti-inflammatory mediators. The uncontrolled inflammatory mediators circulate through the bloodstream and impact the kidneys, resulting in hemodynamic disturbances within the renal region, endothelial dysfunction in renal vasculature, and mitochondrial dysfunction, culminating in renal impairment as evidenced by elevated creatinine and urea levels (12). Therefore, we propose that RCD plays a significant role in the systemic inflammatory response and renal inflammatory response induced by sepsis, with underlying mechanisms that exhibit notable similarities.

Thus, targeting RCD is considered a promising strategy for the management of SA-AKI. RCD refers to forms of cell death regulated by various biomolecules, in contrast to accidental cell death (ACD). Current research indicates that inflammation-associated RCD primarily includes necroptosis, pyroptosis, autophagic cell death, and ferroptosis (13). These RCD modalities have been shown to play significant roles in the pathogenesis and progression of sepsis-induced renal injury. The following sections will provide a detailed overview of these RCD types (14–17).

### 2.1 Pyroptosis in SA-AKI

Pyroptosis represents a distinctive form of regulated cell death, marked by cell swelling and membrane rupture, resulting in the release of intracellular contents and the activation of a potent inflammatory response (18). This process, mediated by gasdermin proteins, is integral to innate immunity and plays a crucial role in managing infections and inflammatory responses. In the context of sepsis and sepsis-induced organ damage, pyroptosis functions as a defensive mechanism aimed at clearing intracellular pathogens (19). Recent research underscores its relevance as a focal point of investigation. For example, Li et al. reported increased expression of nucleotide- binding oligomerization domain, leucine- rich repeat and pyrin domain- containing 3(NLRP3) and heightened inflammatory responses in renal tissue of mice with CLP-induced sepsis (20). Similarly, early stages of SA-AKI have been associated with elevated levels of pyroptosis markers, including NLRP3, Caspase-1, and gasdermin D (GSDMD) (21). This section provides a comprehensive review of the canonical pathways and key proteins involved in pyroptosis in SA-AKI, with the objective of offering evidence to support targeting pyroptosis in the treatment of sepsis-associated renal injury.

#### 2.1.1 Classical pathways of pyroptosis in SA-AKI

**Figure 1:**
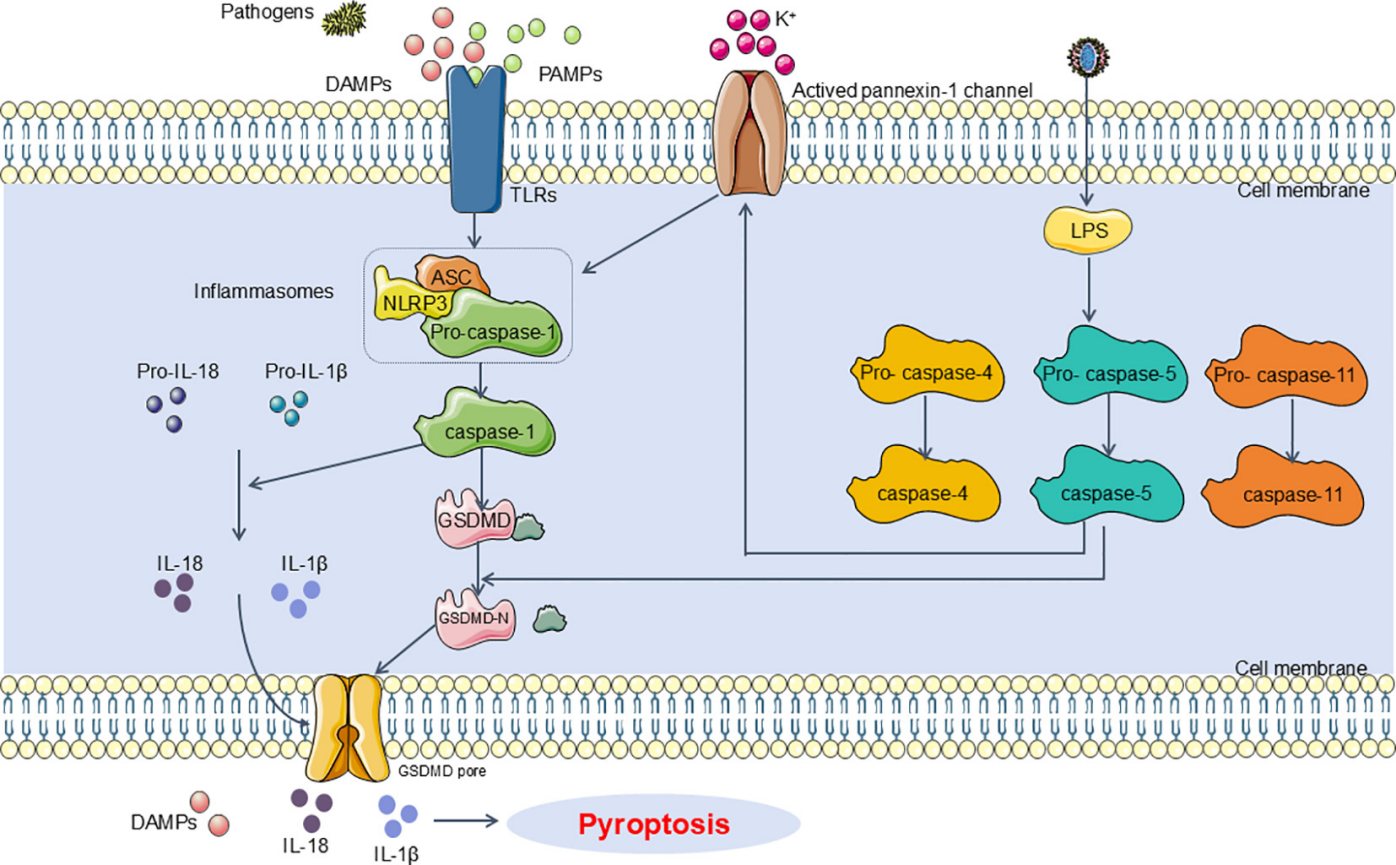
Main pathway of pyroptosis in SA-AKI. Pyroptosis can be categorized into two types depending on whether they are caspase1 dependent or not. In caspase1 dependent pyroptosis, the process is initiated by the assembly of inflammasomes. In caspase-1 non-dependent pyroptosis can be triggered by the interaction between caspase4, caspase5, or caspase11 (depending on the species) and LPS. DAMPs, damage-associated molecular patterns; PAMPs, Pathogen-associated molecular patterns; TLRs, Toll-like receptors; NLRP3, NOD-like receptor protein 3; ASC, apoptosis-associated speck-like protein containing CARD; LPS, Lipopolysaccharides.

In the pathogenesis of SA-AKI, pyroptosis is primarily mediated by inflammasome activation, which engages certain caspase family proteins to cleave gasdermin proteins. This cleavage activates gasdermin proteins, which then translocate to the membrane to form pores. The formation of these pores leads to cell swelling, cytoplasmic leakage, and ultimately cell membrane rupture, resulting in pyroptotic cell death. This process is categorized into two classical pathways based on caspase dependency: the Caspase-1-dependent pyroptosis pathway and the Caspase-1-independent pyroptosis pathway. In the Caspase-1-dependent pyroptosis pathway, upon the invasion of various pathogens, inflammasomes such as NLRP3, Nucleotide-binding oligomerization domain, leucine-rich repeat and caspase recruitment domain-containing 4(NLRC4), absent in melanoma 2(AIM2), and Pyrin detect and respond to these signals, leading to their activation (22). These inflammasomes recruit the adaptor protein apoptosis-associated speck-like protein (ASC), which then associates with Pro-Caspase-1 to activate Caspase-1. Activated Caspase-1 cleaves Gasdermin D (GSDMD), exposing the N-terminal domain of GSDMD, which binds to phospholipids on the cell membrane, forming pores that release intracellular contents and induce pyroptosis (23). Concurrently, activated Caspase-1 also cleaves and activates the precursors of IL-1β and IL-18. The mature IL-1β and IL-18 are then released extracellularly, further amplifying the inflammatory response (24). In the Caspase-1-independent pathway of pyroptosis, exposure to lipopolysaccharides (LPS) results in the direct activation of human Caspase-4 and Caspase-5, as well as mouse Caspase-11 (25). These caspases bind to LPS, leading to their activation and subsequent cleavage of GSDMD. The cleavage of GSDMD exposes its N-terminal domain, which initiates the pyroptotic process. Additionally, the activated Caspase-4/5/11 further induces the activation of the Pannexin-1 channel, facilitating the release of potassium ions (K+) into the extracellular milieu. This potassium efflux subsequently activates the NLRP3 inflammasome, which in turn activates Caspase-1, thus engaging the Caspase-1-dependent pyroptosis pathway (26) (**Figure 1**).

#### 2.1.2 Key factors of pyroptosis in SA-AKI

Similar to its role in other pathological processes, pyroptosis in SAKI involves key proteins including the NLRP3 inflammasome, GSDMD, and pro-inflammatory cytokines. Specifically, the activation of the NLRP3 inflammasome, the formation of GSDMD pores, and the secretion of pro-inflammatory cytokines are considered critical events in the occurrence of pyroptosis in SAKI.

##### 2.1.2.1 Gasdermin-D

GSDMD, a member of the gasdermin (GSDM) family, is the first identified executioner of pyroptosis. Research indicates that the human genome encodes six GSDM family members (GSDMA, GSDMB, GSDMC, GSDMD, GSDME, and DFNB59), while the mouse genome encodes ten members (GSDMA1, GSDMA2, GSDMA3, GSDMC, GSDMC2, GSDMC3, GSDMC4, GSDMD, GSDME, and DFNB59). Like other family members, GSDMD features a cytotoxic N-terminal domain and a C-terminal inhibitory domain connected by a flexible linker. Upon cleavage of the C-terminal domain of GSDMD, the N-terminal fragment (GSDMD-N) is recruited to the cell membrane, interacting with lipids to form an intermediate structure known as a pre-pore. Following a conformational rearrangement, the pre-pore assembles into a crescent-shaped oligomer, which subsequently transitions into a pore-like structure, eventually forming a membrane pore. Electron microscopy reveals that the inner diameter of GSDMD-N pores ranges from 10 to 15 nm, allowing the passage of certain pro-inflammatory cytokines such as IL-1β and IL-18, thereby triggering pyroptosis (27, 28). Yang et al. observed that in a septic mouse model, GSDMD-/- mice exhibited reduced organ damage compared to wild-type mice, indicating a mitigated septic response (29).

##### 2.1.2.2 NOD-like receptor thermal protein domain associated protein 3 Inflammasome

The NLRP3 inflammasome is a substantial multiprotein complex, approximately 700,000 Da in molecular weight, consisting of NLRP3, the adaptor protein ASC, and the effector protein caspase-1. Its assembly necessitates interactions among the NLRP3 receptor, the ASC adaptor, and pro-caspase-1. In a resting state, NLRP3 is kept in an auto-inhibited configuration. The recognition of PAMPs or DAMPs triggers a conformational change in NLRP3, lifting the auto-inhibition. This process allows the N-terminal PYD domain of NLRP3 to recruit ASC adaptor proteins, which also contain a PYD domain. The subsequent recruitment of pro-caspase-1 is mediated by the caspase recruitment domain (CARD) domain of ASC, resulting in the assembly of the inflammasome complex. ASC, which consists of PYD and CARD domains, is predominantly found in the nucleus of human monocytes/macrophages (30). During stress conditions, ASC rapidly relocates to the cytoplasm, linking NLRP3 with pro-caspase-1 and promoting the activation of the NLRP3 inflammasome. Caspase-1, also referred to as IL-1β converting enzyme, acts as the effector protein of the NLRP3 inflammasome (31). It is activated through the autocatalytic cleavage of its precursor, pro-caspase-1, producing active caspase-1. Caspase-1’s primary role is to convert pro-IL-1β and pro-IL-18 into their mature forms, IL-1β and IL-18, respectively. Several clinical studies have indicated elevated levels of NLRP3, GSDMD, IL-1β, and IL-18 in sepsis patients (32–34). In models of SA-AKI, such as mice injected with LPS and HK-2 cells treated with LPS *in vitro*, activation of the NLRP3 inflammasome has been implicated in the induction of pyroptosis in renal cells. The NLRP3 inflammasome exerts indirect regulation of pyroptosis via the caspase-1, reactive oxygen species (ROS), and Panx1 signaling pathways (35). Additionally, the research highlights that Panx1 inhibition can attenuate NLRP3 inflammasome activation, leading to decreased levels of IL-1β, IL-6, and TNF-α in SI-AKI, thereby mitigating septic kidney injury (35).

##### 2.1.2.3 Pro-inflammatory cytokines

Pro-inflammatory cytokines such as IL-1β and IL-18 can affect renal endothelial cells, leading to alterations in microcirculation and ultimately resulting in SA-AKI. Research indicates that endothelial dysfunction induced by inflammation and oxidative stress, along with coagulation disturbances and glycocalyx degradation, may cause microcirculatory impairment even when large vessels remain intact (8, 36, 37). Functional studies using *in vivo* video microscopy in LPS-induced septic mice have demonstrated impaired blood flow in peritubular capillaries, leading to tubular stress and renal damage (38).

In summary, the central events in pyroptosis encompass NLRP3 inflammasome activation, GSDMD pore formation, and the release of pro-inflammatory cytokines. The NLRP3 inflammasome is activated by various danger signals, including PAMPs, DAMPs, and metabolites associated with metabolic disturbances (such as ATP and K^+^). This activation leads to GSDMD pore formation and the subsequent release of IL-1β and IL-18 into the bloodstream. These events initiate extensive inflammatory and immune responses, culminating in sepsis and SA-AKI. Consequently, targeting pyroptosis inhibition emerges as a promising therapeutic approach for managing SA-AKI.

### 2.2 Necroptosis in SA-AKI

Necroptosis represents a form of regulated cell death that is activated when apoptosis is obstructed. It is initiated by extracellular stimuli, such as interactions between death receptors and their ligands, or by intracellular signals, including pathogen-derived nucleic acids (39). Unlike apoptosis, necroptosis is marked by a more disordered and violent cellular demise, involving immune-mediated destruction of the host’s own cells. Necroptotic cells display distinctive necrotic morphology, including plasma membrane rupture, cellular and organellar swelling, and subsequent disintegration. Concurrently, necroptosis instigates a significant inflammatory response marked by widespread infiltration and activation of inflammatory cells (40, 41). Emerging research underscores the critical role of necroptosis in the mechanisms underlying inflammation and the pathogenesis of infectious diseases (42, 43). In sepsis, the mechanism involves systemic immune activation by pathogenic microorganisms, which precipitates severe and prolonged inflammatory responses (44, 45). Thus, necroptosis can be regarded as an initiating “cell death storm,” which may lead to either acute or chronic inflammatory state. Molecular mediators targeting necroptosis present promising therapeutic targets for sepsis and associated organ damage. In this section, we will elucidate the role of necroptosis in SA-AKI by detailing the classic pathways and key molecules involved in necroptotic cell death. This will provide insights into the potential of targeting necroptosis as a future therapeutic strategy for SA-AKI.

#### 2.2.1 Classical pathways of necroptosis in SA-AKI

**Figure 2:**
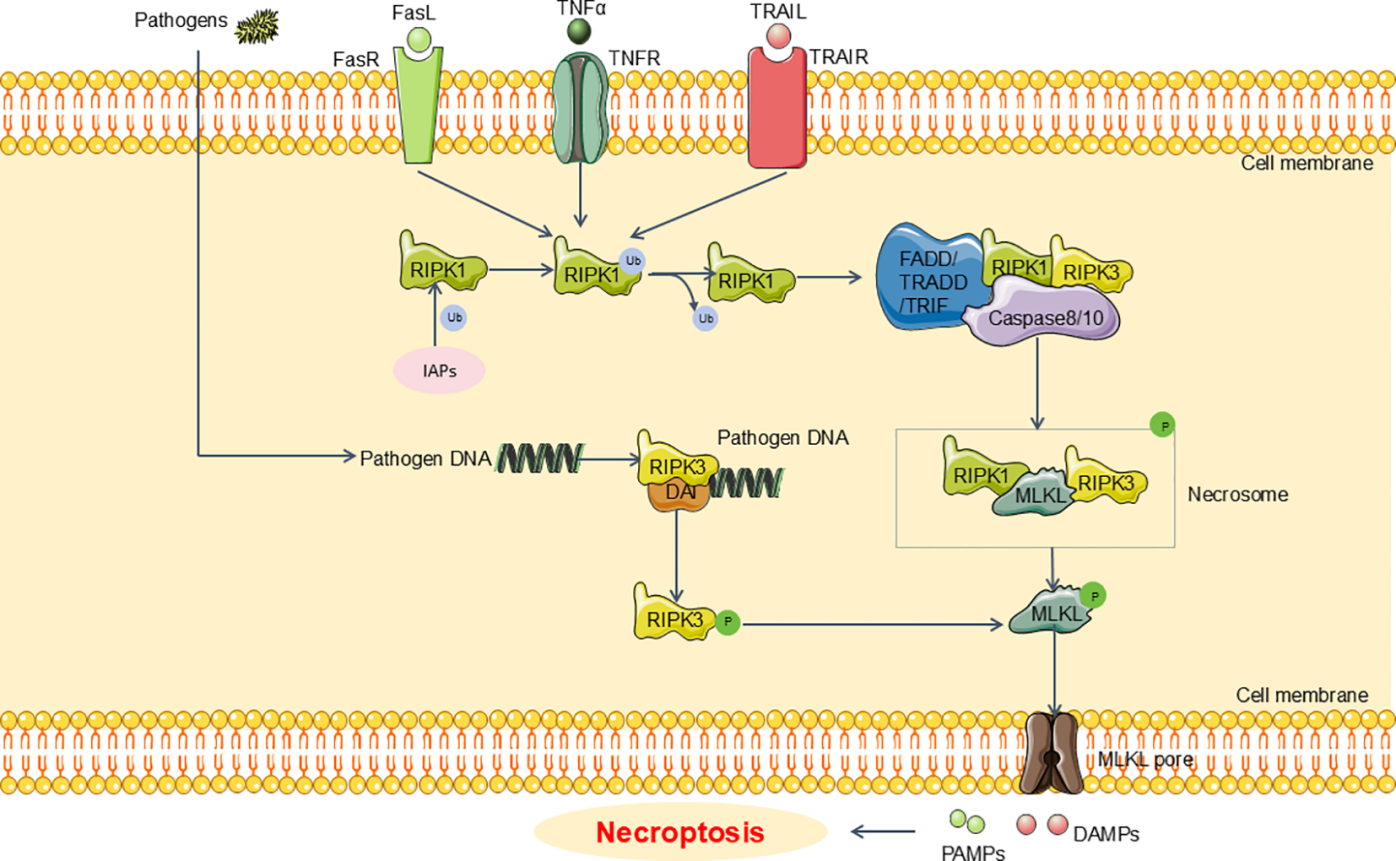
Main pathway of necroptosis in SA-AKI. Upon engagement of these receptors with their ligands, adaptor proteins including FADD, TRADD, and TRIF are recruited. Moreover, RIPK1 undergoes deubiquitination by CYLD, facilitating the recruitment of RIPK3. The RIPK1/RIPK3 complex then phosphorylates MLKL. Activated MLKL oligomerizes to form necrosomes, which generate large pores in the plasma membrane. This pore formation allows for the uncontrolled release of intracellular contents, ultimately culminating in necroptosis. FADD, FAS-associated death domain;TRADD, tumor necrosis factor receptor-associated death domain; TRIF, TIR domain-containing adaptor inducing interferon-β; CYLD, cylindromatosis; RIPK1, receptor-interacting serine/threonine protein kinases1; RIPK3, receptor-interacting serine/threonine protein kinases3; MLKL, mixed lineage kinase domain-Like; IAPs, inhibitor of apoptosis proteins.

Recent research has elucidated that the canonical pathway of necroptosis involves the RIPK1/RIPK3/MLKL signaling axis. In the context of sepsis and sepsis-induced renal injury, necroptosis is predominantly initiated downstream of death domain receptors (such as tumor necrosis factor receptor(TNFR) and Fas) and Toll-like receptors (TLR4 or TLR3) (46–48). Upon engagement of these receptors with their ligands, adaptor proteins including FAS-associated protein with death domain (FADD), tumor necrosis factor receptor-associated death domain protein (TRADD), and TIR domain-containing adaptor inducing interferon-β (TRIF) are recruited. These adaptors interact with receptor-interacting serine/threonine protein kinases1(RIPK1) and caspase-8 or -10, leading to a cascade of subsequent molecular events (49, 50). Typically, RIPK1 is kept in a non-functional state through ubiquitination by inhibitor of apoptotic proteins (IAPs). However, in the presence of death signals, RIPK1 undergoes deubiquitination by cylindromatosis (CYLD), facilitating the recruitment of receptor-interacting serine/threonine protein kinases 3(RIPK3) (51, 52). The RIPK1/RIPK3 complex then phosphorylates mixed lineage kinase domain-Like (MLKL) (53). Activated MLKL oligomerizes to form necrosomes, which generate large pores in the plasma membrane. This pore formation allows for the influx of ions, leading to cellular swelling, membrane rupture, and the uncontrolled release of intracellular contents, ultimately culminating in necroptosis (54). Recent investigations have revealed that pathogenic microorganisms can release cytoplasmic DNA, which subsequently engages the DNA-dependent activator of IFN regulatory factor (DAI) (55, 56). This interaction facilitates the recruitment of RIPK3, thereby bypassing RIPK1 and directly activating MLKL (55, 56). This process results in the formation of necrosomes and contributes to necroptosis (55, 56). Emerging evidence suggests that targeting necroptosis may offer therapeutic benefits for sepsis-induced renal injury. For example, Sun et al. have shown that Dexmedetomidine (Dex) can alleviate LPS-induced necroptosis in HK2 cells, leading to an attenuation in renal damage in sepsis models (57) (**Figure 2**).

#### 2.2.2 Key factors of necroptosis in SA-AKI

In SA-AKI, the principal proteins implicated in necroptosis, similar to those in established necroptotic pathways, encompass RIPK1, RIPK3, and MLKL.

##### 2.2.2.1 Receptor-interacting protein kinase 1 and receptor-interacting protein kinase 3

RIPK1 and RIPK3, both serine/threonine kinases, function as essential regulators in the execution of necroptotic cell death. Clinical investigations have revealed that RIPK3 levels are markedly increased across all time points in patients with severe sepsis and septic shock compared to those with sepsis alone (58). Additionally, RIPK3 levels exhibit a positive correlation with sequential organ failure assessment (SOFA) scores and procalcitonin levels (58). Recent clinical trials have established necroptosis as a prognostic marker for mortality in sepsis patients, with RIPK3 levels being utilized as a biomarker for evaluating necroptotic processes (59). Based on clinical trials, researchers have conducted molecular-level studies and found that the necroptosis marker RIPK3 is positively correlated with mortality and organ dysfunction in sepsis (60). Furthermore, in various injury models, Necrostatin-1 (Nec-1) has been shown to inhibit RIPK1 kinase activity, thereby suppressing necroptosis (61, 62). In the Cecal Ligation and Puncture (CLP) mouse model, renal gut-derived 4-hydroxyphenylacetic acid (4-HPA) enhances the interaction between apoptosis repressor with caspase recruitment domain (ARC) and RIPK1 by upregulating ARC protein expression (16). This interaction inhibits necroptosis in renal tubular epithelial cells, reduces CLP-induced increases in serum creatinine, urea nitrogen, and cystatin C, and improves overall survival rates (16).

##### 2.2.2.2 Mixed lineage kinase domain-like protein

MLKL, a key downstream effector in the RIPK1/RIPK3/MLKL signaling pathway, acts as a principal mediator of necroptosis. Upon activation, MLKL translocate to the cell membrane, causing membrane rupture and the release of danger signals (13). Newton et al. demonstrated that the simultaneous loss of MLKL and caspase-8 substantially inhibited TNF-induced cytokine storms, hypothermia, and morbidity, and alleviated renal damage in LPS-induced sepsis mouse models (63). Furthermore, in LPS-induced sepsis models, the administration of Necrosulfonamide (NSA) to inhibit MLKL activity effectively suppressed necroptosis (64).

In summary, the inhibition of necroptosis mediated by the RIPK1/RIPK3/MLKL signaling pathway confers protective effects in the context of sepsis. Nonetheless, necroptosis is not entirely detrimental to the organism. It is instrumental in the removal of excess lymphocytes, a process critical for maintaining lymphocyte homeostasis (65). Moreover, in response to bacterial and viral infections, eukaryotic cells may engage in necroptosis to curtail pathogen replication (66). Therefore, additional research is warranted to elucidate the role of necroptosis in sepsis and SA-AKI.

### 2.3 Autophagy in SA-AKI

Emerging evidence underscores the significant role of autophagy in acute kidney injury associated with sepsis. Autophagy, regulated by autophagy-related genes (Atg) in eukaryotic cells, involves the lysosomal degradation of cytoplasmic proteins and damaged organelles (67). This process is essential for cellular protection, survival under nutrient scarcity, and response to cytotoxic stress. Autophagy encompasses both basal and stress-induced forms. Basal autophagy, operational under normal physiological conditions, contributes to cell growth, development, and the maintenance of cellular homeostasis by regulating the synthesis, degradation, and recycling of cellular components. Conversely, excessive autophagy can precipitate metabolic stress, degradation of cellular components, and potentially lead to cell death (68). The process of autophagy involves the reorganization of cellular membranes and progresses through four key stages: initiation, formation of isolation membranes and autophagosomes, fusion of autophagosomes with lysosomes, and the subsequent degradation of autophagosomes (69). Autophagy is integral to various physiological and pathological processes, including cellular homeostasis, aging, immunity, tumorigenesis, and neurodegenerative diseases.

Autophagy plays a dual role in various human diseases, both mitigating and exacerbating pathological processes. It is currently understood that autophagy is activated during the early phases of sepsis but becomes depleted in later stages (70). Hsiao et al. reported that in a CLP mouse model, the levels of the autophagy marker LC3-II were elevated 3 hours post-CLP surgery, but declined between 9 and 18 hours post-surgery, which was associated with deteriorating renal function (71). Therefore, the inhibition of autophagy can precipitate proximal tubular dysfunction and contribute to the development of SA-AKI.

**Figure 3:**
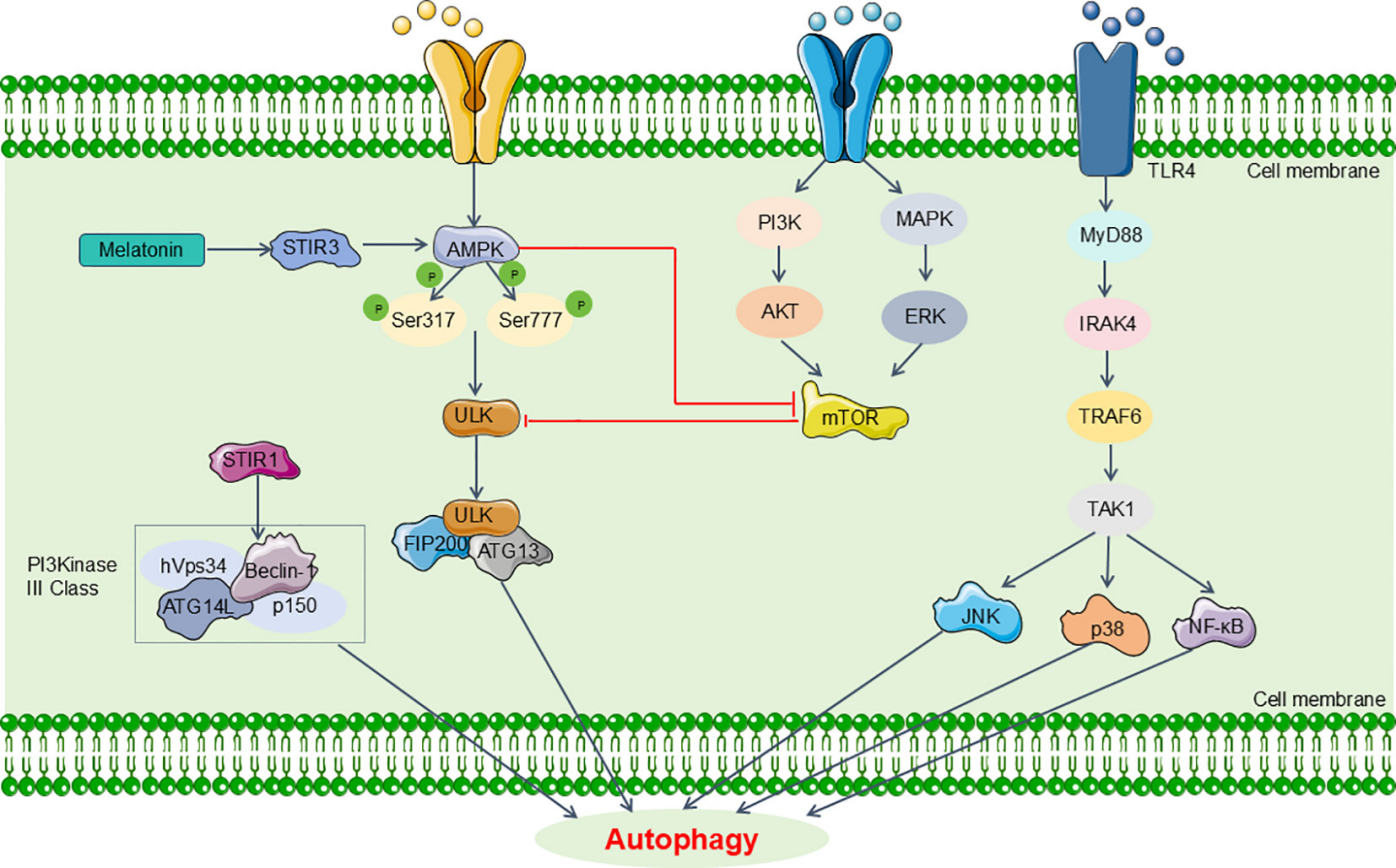
Main pathway of autophagy in SA-AKI. The signaling pathways related to autophagy in SA-AKI mainly include MAPK/mTOR, CaMK, SIRT and TLR4 signaling, which might be promising candidates for targeted therapeutic strategies aimed at SA-AKI in future clinical applications. MyD88, myeloid differentiation primary response 88; IRAK4, IL-1 receptor-associated kinase-4; TRAF6, tumor necrosis factor receptor- associated factor 6; TAK1, transforming growth factor-β-activated kinase 1.

In the context of sepsis, autophagy can have divergent effects depending on the organ system. Specifically, it has been found to offer protective benefits in the lungs, heart, kidneys, and brain. Conversely, in skeletal muscle, autophagy may exacerbate tissue damage (72). Recent research highlights its critical involvement in the regulation of renal pathophysiology (68). Some investigations have indicated that activating autophagy may help mitigate SA-AKI. For instance, Leventhal et al. demonstrated in an LPS-induced sepsis mouse model that Atg7 gene knockout resulted in aggravated tubular damage and significantly elevated BUN levels (73). Additionally, they observed increased IL-6 and phosphorylated signal transducer and activator of transcription 3 (STAT3) expression in the kidneys of Atg7 knockout mice compared to controls post-LPS treatment, suggesting that autophagy activation may alleviate SA-AKI (73). However, alternative studies propose that autophagy activation could exacerbate renal damage during sepsis. Wu et al., using a CLP sepsis mouse model, reported that autophagy inhibition with 3-MA led to decreased Beclin1 and LC3-II/I levels but caused p62 accumulation and worsened renal damage (74). This implies that impaired autophagy exacerbates acute kidney injury in sepsis models (74). Nevertheless, multiple researches predominantly support the view that dysregulated autophagy contributes to pathological damage, with SA-AKI typically involving suppressed renal cell autophagy (75, 76). Ongoing investigations focus on specific proteins and signaling pathways or factors related to autophagy in SA-AKI (**Figure 3**).

#### 2.3.1 AMPK and mTOR signaling pathways of autophagy regulation in SA-AKI

As an initiator of autophagy, AMPK activates ULK1 by phosphorylating Ser 317 and Ser 777 of ULK1, thereby initiating the autophagy process and attenuating SA-AKI (77). Additionally, AMPK negatively regulates mTOR activity. Consequently, AMPK activation suppresses mTOR activity, promotes autophagy, and limits the excessive production of pro-inflammatory cytokines, thereby mitigating kidney damage caused by sepsis (11, 78). Similarly, inhibition of AMPK activation by compound C can block autophagy and exacerbate CLP-induced kidney injury (11). These findings indicate that the AMPK/mTOR signaling pathway is involved in SA-AKI.

#### 2.3.2 Sirtuins family of autophagy regulation in SA-AKI

Sirtuins (SIRTs) represent a family of nicotinamide adenine dinucleotide (NAD)^+^-dependent histone deacetylases with seven distinct isoforms (SIRT1-SIRT7). These enzymes play critical roles in a range of pathological and physiological processes, such as inflammation, fibrosis, atherosclerosis, energy metabolism, age-related diseases, and cancer (79, 80). Specifically, activation of SIRT1 leads to the deacetylation of Beclin1, which in turn promotes autophagy and mitigates kidney injury associated with sepsis (80). Autophagy activation via the AMPK/SIRT1 signaling pathway has been shown to mitigate sepsis-induced renal injury (77). Additionally, SIRT3 plays a role in sepsis-associated renal damage. In a model of CLP-induced SA-AKI, overexpression of SIRT3 accelerates autophagy, attenuating tubular cell apoptosis and the accumulation of pro-inflammatory cytokines (81). These outcomes are mediated through the AMPK/mTOR pathway (81). Consequently, SIRT3 emerges as a potential therapeutic target for treating SA-AKI. For instance, melatonin can mitigate sepsis-induced acute kidney injury by promoting mitochondrial autophagy through SIRT3-mediated deacetylation of mitochondrial transcription factor A (TFAM) (82). SIRT6 is also involved in the regulation of autophagy. Zhang et al. demonstrated that LPS upregulates the expression of the SIRT6 gene in HK-2 cells within a murine model of acute kidney injury (83). Their findings further revealed that SIRT6 overexpression not only mitigates LPS-induced apoptosis in HK-2 cells but also promotes autophagy (83). Conversely, silencing of SIRT6 leads to increased secretion of TNF-α and IL-6 in HK-2 cells and diminished autophagy, which contributes to the exacerbation of LPS-induced renal injury (83).

#### 2.3.3 Calcium/calmodulin dependent protein kinase IV/mammalian target of rapamycin signaling pathway of autophagy regulation in SA-AKI

Recent research has elucidated the role of Calcium/calmodulin-dependent protein kinase (CaMK) in SA-AKI. In a murine sepsis model, Zhang et al. demonstrated that CaMKIV augments autophagy in macrophages and renal tissues by inhibiting the serine phosphorylation of glycogen synthase kinase-3β (GSK-3β) and obstructing the recruitment of F-box and WD repeat domain containing 7 (FBXW7) (84). This inhibition prevents the ubiquitin-proteasome-mediated degradation of mammalian target of rapamycin (mTOR) (84). This observation contradicts the established view that mTOR inhibition consistently promotes autophagy. Furthermore, the results indicate that CaMKIV can regulate autophagy via mechanisms that are independent of mTOR suppression, underscoring its pivotal role in modulating autophagy in the context of LPS-induced acute kidney injury.

#### 2.3.4 Toll-like receptor4 signaling pathway of autophagy regulation in SA-AKI

Toll-like receptors (TLRs) constitute a superfamily of single-pass transmembrane receptors expressed across various tissues and numerous cell types, functioning as pattern recognition receptors that identify microbial PAMPs. This family includes TLR1-11. Among them, TLR4 has been extensively studied in relation to autophagy. The activation of the TLR4 signaling pathway originates from the Toll/IL-1 receptor (TIR) domain in the cytoplasm, which interacts with the TIR domain-containing adaptor protein MyD88. Upon ligand stimulation, MyD88 recruits IL-1 receptor-associated kinase-4 (IRAK-4) to TLRs through interactions involving two death domains. IRAK-1 is subsequently phosphorylated and activated, leading to its association with TRAF6. This interaction activates the IKK complex, which in turn triggers the activation of MAP kinases (JNK, p38 MAPK) and NF-κB, ultimately contributing to the regulation of autophagy. Recent research underscores the critical role of TLR4 in autophagy regulation during sepsis. Li et al. demonstrated in a sepsis mouse model that TLR4 inhibition resulted in reduced levels of platelet Ca2+, mitoSOX fluorescence, NOX2 expression, and a diminished LC3II/LC3I ratio (85). These changes were linked to attenuated autophagy and a significant 80% reduction in septic renal injury (85). Furthermore, a separate study involving renal TECs from C57BL/10 mice, which are deficient in functional TLR4, revealed that LPS incubation did not induce autophagy in these TECs compared to wild-type controls. This observation highlights the protective role of RTEC autophagy against endotoxin-induced damage and underscores the necessity of TLR4 signaling for autophagy induction in TECs (73). Together, these findings might affirm that TLR4 is indispensable for LPS-induced autophagy in TEC cells.

Additionally, it is noteworthy that Receptor Interacting Protein Kinase 3 (RIP3) and its associated signaling pathways have been implicated in tubular damage and renal dysfunction during SA-AKI. Li et al. reported in an LPS-induced sepsis mouse model that activation of RIP3 disrupts the TFEB-lysosomal pathway, obstructing degradation of the autophagosome marker LC3II and the autophagy substrate p62 (86). These observations propose RIP3 as a promising therapeutic target for mitigating and managing septic AKI (86).

In conclusion, autophagy is critically involved in the regulation of both the initiation and progression of SA-AKI. Classical signaling pathways associated with autophagy, such as MAPK, mTOR, CaMK, and TLR4, are promising candidates for targeted therapeutic strategies aimed at mitigating septic kidney damage in future clinical applications.

### 2.4 Ferroptosis in SA-AKI

Ferroptosis is a novel form of iron-dependent programmed cell death distinct from apoptosis and autophagy (87). In physiological conditions, an oxidative system involving iron ions, the Fenton reaction, and ROS is balanced by antioxidant mechanisms that include the cystine/glutamate antiporter (system Xc−), glutathione peroxidase 4 (GPX4), and glutathione (GSH) within the system Xc−/GSH/GPX4 axis, along with other recently identified pathways. This balance is crucial for maintaining cellular and organismal homeostasis. However, in pathological contexts such as infection, inflammation, or cancer, this equilibrium can be disrupted, leading to the activation of ferroptosis and its associated adverse consequences (88). The primary mechanism of ferroptosis involves the catalytic oxidation of polyunsaturated fatty acids on cell membranes, mediated by divalent iron or lipoxygenases, leading to lipid peroxidation and subsequent cell death. The essence of ferroptosis lies in the depletion of glutathione and the reduced activity of GPX4. This reduction impairs the ability to metabolize lipid peroxides via the GPX4-catalyzed glutathione reduction reaction, triggering the oxidation of lipids by divalent iron and the generation of reactive oxygen species, thereby driving ferroptosis. The occurrence and execution of ferroptosis are influenced by various metabolic processes, including iron homeostasis, amino acid metabolism, and lipid peroxidation. Previous study has elucidated that molecules like coenzyme Q also modulate ferroptosis sensitivity (89). In ferroptosis, cellular mitochondria undergo shrinkage, increased membrane density, and diminished cristae, while nuclear morphology remains relatively intact. This process is characterized by heightened intracellular lipid peroxidation and elevated ROS levels. The hallmark of ferroptosis is the production of lipid peroxides, which compromise cell membrane integrity and induce cell death (13). ROS imbalance, coupled with insufficient antioxidants or free radical scavengers, precipitates oxidative stress and activates various pro-inflammatory factors, including NF-kB and hypoxia-inducible factor-1α (90). Moreover, Arachidonic acid (AA), a principal polyunsaturated fatty acid (PUFA) in cellular membranes, is metabolized via three pathways into bioactive pro-inflammatory mediators, which are pivotal in the inflammatory processes underlying renal injury (91). Consequently, PUFAs and their metabolic enzymes are recognized as critical modulators of major inflammatory pathways. Ferroptotic cells release DAMPs, which serve as pro-inflammatory danger signals and facilitate the release of inflammatory mediators, including HMGB1, IL-33, and other yet-to-be-identified factors (90). Overall, the interplay between ferroptosis and inflammation can initiate and sustain a self-reinforcing cycle, leading to enhanced inflammation and tissue damage. Ferroptosis is involved in the pathophysiological processes of multiple organ systems, encompassing the nervous, urinary, hepatic, and cardiovascular systems (92–94).

Extensive research has elucidated that ferroptosis primarily occurs under conditions characterized by metabolic dysregulation and oxidative stress (95). Ferroptosis in immune or other cells affects the body’s immune response, thereby contributing to the onset and progression of sepsis (95). Bacterial infections are a major precipitating factor for sepsis. For example, during the bacterial infection phase in severe pancreatitis, ferroptosis within intestinal epithelial cells can disrupt the intestinal barrier, thereby allowing the translocation of pathogenic intestinal bacteria and toxins into the systemic circulation and extraluminal tissues (96). On the other hand, certain studies have observed that, early in the immune response, there is a marked increase in iron and lipid peroxidation within macrophages (97). Ferroptosis inducers such as RSL3, salazosulfapyridine, and acetaminophen have been shown to enhance bacterial clearance by macrophages (97). These findings suggest that ferroptosis may act as a double-edged sword in the context of sepsis, as it can both exacerbate bacterial invasion and sepsis progression, while also contributing to the elimination of pathogens by immune cells. However, the prevailing view remains that ferroptosis induces inflammatory dysregulation, thereby contributing to sepsis and associated organ damage (98, 99). Renal inflammatory dysregulation is a crucial mechanism in SA-AKI, with ferroptosis closely associated with these inflammatory disturbances. Recent research has increasingly explored the link between ferroptosis and SA-AKI (100, 101). For example, Luo et al. demonstrated in an LPS-induced sepsis kidney injury mouse model that DEX alleviates ferroptosis through the Keap1-Nrf2/HO-1 signaling pathway, thereby modulating oxidative stress and providing a therapeutic approach for SA-AKI (102). This evidence highlights the pivotal role of ferroptosis in the development and progression of SAKI. This section will examine ferroptosis in the context of SA-AKI, with a focus on its classical pathways and key factors.

#### 2.4.1 Classical pathways of ferroptosis in SA-AKI

**Figure 4:**
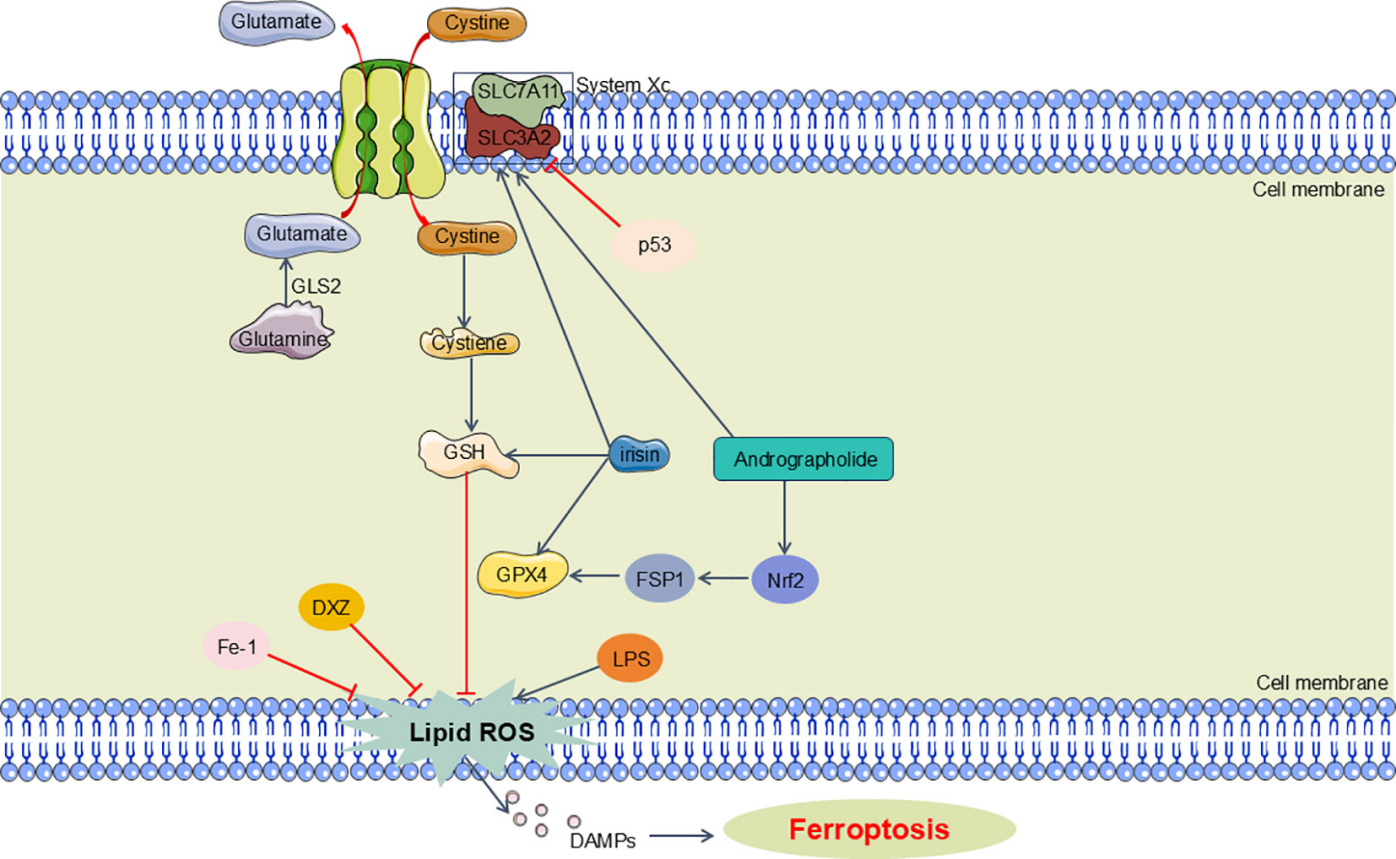
Main pathway of ferroptosis in SA-AKI. Ferroptosis can be triggered via two primary mechanisms: the exogenous/transport-dependent pathway and the endogenous/enzyme-regulated pathway. In the exogenous/transport-dependent pathway, System xc- imports extracellular cystine into the cell, essential for glutathione synthesis. Glutathione is a key reductive cofactor for GPX4, which protects cells from oxidative stress. The endogenous pathway that involves critical enzymes such as GPX4FSP1. GLS2, glutaminase 2; GSH, glutathione; GPX4, glutathione peroxidase 4; FSP1, ferroptosis suppressor protein 1; DXZ, dexrazoxane; Fe-1, Ferrostatin-1.

In the progression of SA-AKI, ferroptosis can be triggered via two primary mechanisms: the exogenous/transport-dependent pathway and the endogenous/enzyme-regulated pathway. In the exogenous/transport-dependent pathway, conditions such as infection, stress, or inflammation can result in the inhibition of membrane transport proteins, notably the cystine/glutamate antiporter (System xc-). System xc- normally functions to import extracellular cystine into the cell, which is crucial for the synthesis of glutathione. Glutathione serves as a reductive cofactor for GPX4. A decrease in glutathione levels leads to reduced GPX4 activity, impairing the cellular antioxidant defense system and promoting ferroptosis. Furthermore, the activation of iron transport proteins, such as transferrin and lactoferrin, can also precipitate ferroptosis (103, 104). Conversely, the endogenous/enzyme-regulated pathway primarily mediates ferroptosis through the suppression of intracellular antioxidant enzyme activity. This pathway involves critical enzymes such as GPX4, ferroptosis suppressor protein 1 (FSP1), and the coenzyme Q10 (CoQ10) pathway, which includes FSP1, CoQ10, and NADPH. Inhibition of these enzymes disrupts the cellular antioxidant defense mechanisms, thereby facilitating the initiation of ferroptosis (103) (**Figure 4**).

#### 2.4.2 Key factors of ferroptosis in SA-AKI

##### 2.4.2.1 Glutathione peroxidase4

Glutathione peroxidase (GPX), a member of a protein superfamily, features cysteine residues in its catalytic site, which serve as redox-active centers essential for facilitating redox reactions (105, 106). Among the eight human GPX isoforms, GPX4 is critically implicated in ferroptosis. It regulates ferroptosis by converting lipid hydroperoxides into benign lipid alcohols, thereby inhibiting lipid peroxidation under normal physiological conditions. Insufficient GPX4 synthesis or inhibited activity results in the accumulation of peroxides within cells, leading to ferroptosis. GPX4 is frequently targeted by various ferroptosis inducers (107). Furthermore, selenocysteine, an amino acid integral to GPX4’s active site, can be downregulated via the mevalonate(MVA) pathway, which attenuates isopentenylpyrophosphate (IPP) levels and impedes the maturation of selenocysteine tRNA, thereby suppressing GPX4 activity and facilitating ferroptosis (108). Research has demonstrated that ROS generated during sepsis can trigger ferroptosis (109). In LPS-induced sepsis mouse models, it has been observed that LPS elevates the levels of ferroptosis-related markers, including prostaglandin-endoperoxide synthase 2 (PTGS2), malondialdehyde (MDA), and lipid ROS, which subsequently leads to ferroptosis and mitochondrial damage. Compounds such as Fe-1 and DXZ have been shown to alleviate this damage (110). GPX4 is a critical factor in the pathophysiology of SA-AKI. Zhang et al. demonstrated in a CLP sepsis mouse model that irisin decreases ROS production, iron levels, and MDA concentrations, while increasing GSH levels and modulating the expression of ferroptosis-associated proteins (such as GPX4 and ACSL4) in the kidneys (111). This modulation inhibits ferroptosis and ameliorates renal injury (111). Furthermore, Zhou et al. reported that in a sepsis mouse model, elevated expression of cation transport regulator 1 (CHAC1) in HK-2 cells, coupled with decreased GPX4 expression, accelerates ferroptosis and worsens renal injury during sepsis (112). Therefore, targeting GPX4 represents a promising therapeutic approach for mitigating sepsis-induced renal damage.

##### 2.4.2.2 Solute carrier family 7, membrane 11

SLC7A11, also known as xCT, is a pivotal protein in ferroptosis, functioning as a cystine/glutamate antiporter. It primarily facilitates the influx of extracellular cystine into cells, thereby aiding in the synthesis of glutathione and bolstering antioxidant defenses (113). The regulation of SLC7A11 expression and activity is integral to controlling ferroptosis. For example, Erastin, a ferroptosis inducer, inhibits System Xc-, leading to decreased intracellular glutathione (GSH) levels and subsequent ferroptosis (13). Similarly, p53-mediated downregulation of SLC7A11 expression impairs cystine uptake through System Xc-, reducing cystine-dependent glutathione peroxidase activity, compromising cellular antioxidant defenses, increasing lipid peroxidation, and inducing ferroptosis (114). Given its role as a primary source of intracellular GSH, SLC7A11 is crucial for mitigating lipid peroxidation and ferroptosis-related septic renal injury. Additionally, andrographolide has been shown to mitigate iron accumulation and lipid peroxidation by upregulating SLC7A11 and GPX4 levels in HK-2 cells via the Nrf2/FSP1 pathway, thus attenuating ferroptosis and alleviating septic acute kidney injury (115).

The precise mechanisms of ferroptosis in SA-AKI remain unclear. Further research is needed to elucidate how ferroptosis can be modulated to improve sepsis and associated organ damage. This will provide a more robust foundation for exploring ferroptosis as a therapeutic target in SA-AKI.

In addition to the aforementioned forms of regulated cell death, NETosis is also recognized as a mechanism involved in the inflammatory processes associated with sepsis and septic kidney injury. Neutrophils are activated in response to microbial or inflammatory stimuli, leading to the release of neutrophil extracellular traps (NETs) (116). During host defense against bacterial and fungal infections, neutrophils exert their protective functions through pathogen phagocytosis, secretion of granules rich in cytotoxic enzymes, or through necrotic processes that release NETs, a phenomenon known as NETosis. NETs consist of a network of DNA structures embedded with histones and antimicrobial proteins, released by activated neutrophils. Once in circulation, NETs can be recruited to sterile or pathogen-induced injury sites by activated endothelial cells, resident immune cells, and damaged epithelial cells. Upon reaching the injury site, receptors on the surface of neutrophils bind to pro-inflammatory signals such as bacterial components, fungal β-glucans, or cytokines, triggering a signaling cascade that enhances neutrophil effector functions, including activation. Activated neutrophils can degranulate to release their granules, internalize and degrade pathogens via phagocytosis, and subsequently release NETs to neutralize invaders (117).

Current research indicates that there are two classical pathways for NETosis: NADPH oxidase 2 (Nox2)-dependent and Nox2-independent NETosis. On one hand, agents such as PMA, LPS, and Pseudomonas aeruginosa induce Nox-dependent NETosis; on the other hand, agents like calcium ionophores (A23187, ionomycin), urate crystals, certain microbes, and ultraviolet light trigger Nox-independent NETosis through different forms of reactive oxygen species (ROS) (118–120). The generated ROS activate specific kinases (such as MAPK, ERK, p38, and JNK) that lead to transcriptional activation and stimulation of myeloperoxidase (MPO). Notably, in the Nox-independent NETosis process, the activation of the nuclear enzyme peptidylarginine deiminase 4 (PAD4) promotes the citrullination of histones (121, 122). The activation of MPO facilitates the translocation of neutrophil elastase (NE) from granules to the nucleus, resulting in chromatin remodeling. Ultimately, this leads to nuclear membrane breakdown and the release of NETs (123–125).

Studies have shown that in sepsis and associated organ dysfunction, NETs play an indispensable role in clearing pathogens from blood and tissues; however, they may also trigger excessive inflammation, causing host tissue damage (126). Therefore, NETs could have dual roles in sepsis and related organ injuries, such as renal damage. Further investigations reveal that during septic events, stressors like infection can induce significant neutrophil recruitment to renal tubules, resulting in tubular necrosis and the release of DAMPs. Extracellularly free or NET-bound histones are considered crucial mediators of renal epithelial cell necrosis, potentially inducing further release of histones as DAMPs (127–129). These DAMPs and other inflammatory mediators further activate neutrophils, promoting NET release and thereby exacerbating surrounding tissue damage. Histones and NETs can enhance tubular necrosis and capillary injury, ultimately leading to renal dysfunction, clinically manifested by elevated creatinine and urea levels (130). Previous study has suggested that the use of PAD4 inhibitors and anti-histone antibodies targeting NET suppression could alleviate associated renal injury (129, 131). However, our understanding of the specific molecular mechanisms linking NETs to SA-AKI remains limited, necessitating further exploration to provide a more robust theoretical foundation for considering NETs as therapeutic targets in septic renal impairment.

## 3 Metabolic reprogramming in SA-AKI

Mitochondria are double-membrane organelles consisting of various regions including the outer membrane, intermembrane space, inner membrane, cristae, and matrix. These organelles are pivotal for cellular energy metabolism, primarily generating adenosine triphosphate (ATP) through oxidative phosphorylation (OXPHOS) to meet cellular energy demands. The distribution of mitochondria varies significantly among different cell types, tissues, and organs. The kidneys, which contain the second highest amount of mitochondria in the body, consume approximately 7% of the body’s daily ATP, with 95%–99% of this energy derived from oxidative metabolism. Due to the varying energy requirements, mitochondrial density and distribution vary within the nephron. Specifically, proximal tubules and the thick ascending limb of the loop of Henle, which are involved in active ion transport, exhibit the highest mitochondrial concentrations. Mitochondrial dysfunction is a vital factor in the pathogenesis of SA-AKI and significantly impacts prognosis. Recent investigations into mitochondrial dysfunction in SA-AKI have concentrated on metabolic reprogramming. The subsequent sections will delve into the role of metabolic reprogramming in septic renal injury.

Metabolic reprogramming refers to the process by which cells adjust their metabolic pathways to meet altered energy demands in response to environmental challenges. This adjustment enables cells to modulate synthetic reactions, thereby adapting to external stressors and acquiring new functional capacities. In the context of sepsis, metabolic reprogramming is a ubiquitous phenomenon affecting nearly all cell types. Specifically, immune cells and certain organ cells often demonstrate the “Warburg effect,” where glycolysis becomes the predominant energy pathway even in the presence of oxygen, as opposed to OXPHOS (132, 133). This shift is crucial for the functional performance of immune cells during sepsis. Notably, this metabolic reprogramming, where glycolysis replaces OXPHOS, has also been documented in renal tubular epithelial cells (134).

### 3.1 Immune dysregulation and metabolic reprogramming in SA-AKI

#### 3.1.1 Moderate metabolic reprogramming can enhance inflammatory responses in SA-AKI

Evidence suggests that the “Warburg effect” has a favorable impact on sepsis and related organ dysfunctions (135). This metabolic adaptation not only facilitates the production of substantial ATP but also generates metabolites that are crucial for maintaining immune cell functionality (135). In the context of sepsis progression, the upregulation of GLUT1 expression is thought to enhance competitive glucose uptake (136, 137). Conversely, the downregulation of GLUT1 results in a notable decrease in glucose uptake and glycolysis, leading to significant impairments in the survival and functionality of effector T cells (Teff) (136, 138). Additionally, the augmentation of glucose uptake and glycolytic flux, along with accelerated ATP synthesis through glycolysis, ensures that ATP levels are adequately maintained to fulfill the energy requirements of activated immune cells, despite the suppression of OXPHOS. Glycolysis produces essential metabolites, including glucose-6-phosphate (G-6-P), dihydroxyacetone phosphate (DHAP), and glyceraldehyde-3-phosphate (G-3-P), which supply both energy and biosynthetic intermediates necessary for immune system activation, thereby enhancing the speed and effectiveness of the immune response. Restricting glycolysis can reduce the inflammatory response (139, 140). For example, in LPS-stimulated bone marrow-derived macrophages (BMDMs), metformin, which does not activate AMPK, decreases LPS-induced IL-1β production at both mRNA and protein levels while increasing the anti-inflammatory cytokine IL-10 (141). Additionally, ROS contribute to LPS-induced IL-1β mRNA production (142). The mechanistic target of rapamycin (mTOR) functions as a pivotal regulator of cellular metabolism. Inhibition of mTOR with rapamycin or its derivatives typically activates downstream factors such as HIF-1α and GLUT1, enhancing glucose uptake and glycolysis (143, 144). Rapamycin also interrupts the release of pro-IL-1β induced by LPS and subsequent IL-1β release triggered by ATP (145). Furthermore, mTOR suppression by rapamycin promotes the differentiation of CD8^+^ T cells, suggesting a potential therapeutic target for modifying immune dysregulation and inflammation (146, 147). Collectively, early metabolic reprogramming in sepsis, characterized by heightened glycolysis, can initiate a protective inflammatory response in host organs.

#### 3.1.2 Excessive metabolic reprogramming can induce immunosuppression in SA-AKI

Excessive glycolysis may induce immunosuppression, compromising both host defense and immune system functionality. This state of immunosuppression is often marked by diminished expression of pro-inflammatory cytokines (e.g., TNF-α, IL-6, CCL2) and T cell-recruiting chemokines, coupled with an upregulation of anti-inflammatory cytokines (e.g., IL-4, IL-10) (148, 149). Consequently, the host’s ability to respond effectively to secondary infections is impaired. The shift from a hyper-inflammatory to a hypo-inflammatory state is associated with a metabolic transition in immune cells from glucose metabolism to fatty acid oxidation. Studies indicate that during immunosuppression, sepsis patients exhibit increased levels of fatty acid transport proteins (e.g., CD36) and carnitine palmitoyltransferase-1 (CPT-1) in their blood leukocytes (150).

In the context of sepsis and SA-AKI, immunosuppression is marked by an enhancement of anti-inflammatory phenotypes and the pivotal role of lactate, a product of glycolysis. Lactate transcends its role as a mere metabolic byproduct and functions as a significant signaling molecule (151, 152). Evidence reveals that lactate acts through lactate-GPR81 and lactate-GPR132 signaling pathways, which are integral to sepsis and organ damage (153–155). Specifically, lactate mitigates inflammation by inhibiting TLR4-mediated signaling via GPR81, thereby facilitating the polarization of M2 macrophages and diminishing LPS-induced NF-κB activation (154–158) Additionally, lactate influences immune cell migration and cytokine production through its export via the monocarboxylate transporter MCT4. It also initiates “stop migration” signals through lactate transporters SLC5A12 (in CD4^+^ T cells) and SLC16A1 (in CD8^+^ T cells) (158).

In conclusion, during sepsis and SA-AKI, metabolic reprogramming in host organs and cells might be an essential aspect of the host’s defensive response. Lactate, generated via glycolysis, plays a role in attenuating inflammation and aiding in the preservation of immune homeostasis. Nonetheless, an overactive glycolytic pathway can result in elevated lactate concentrations, potentially leading to immune suppression and disruption of immune equilibrium.

### 3.2 Cellular energy allocation and metabolic reprogramming in SA-AKI

In SA-AKI, metabolic reprogramming is intricately linked not only to immune dysregulation but also to the reallocation of cellular energy. It is well-documented that infected animals exhibit a shift towards ATP production and a concurrent rise in free fatty acid levels in the bloodstream. This shift is mediated by mitochondrial fatty acid oxidation (FAO), a process regulated by peroxisome proliferators-activated receptor α (PPARα), which is encoded by the NR1C1 gene. Previous research indicates a decrease in PPAR-α levels in both organs and blood during sepsis. Khaliq et al. have shown that sepsis causes deviations in cellular lipid metabolites from their “safe range,” with these deviations correlating with increased mortality (159). Furthermore, Takuma et al. demonstrated that mice lacking PPAR-α have compromised renal function and that diminished PPAR-α signaling exacerbates the incidence of SA-AKI (160).

### 3.3 Metabolic reprogramming of renal cells in SA-AKI

**Figure 5:**
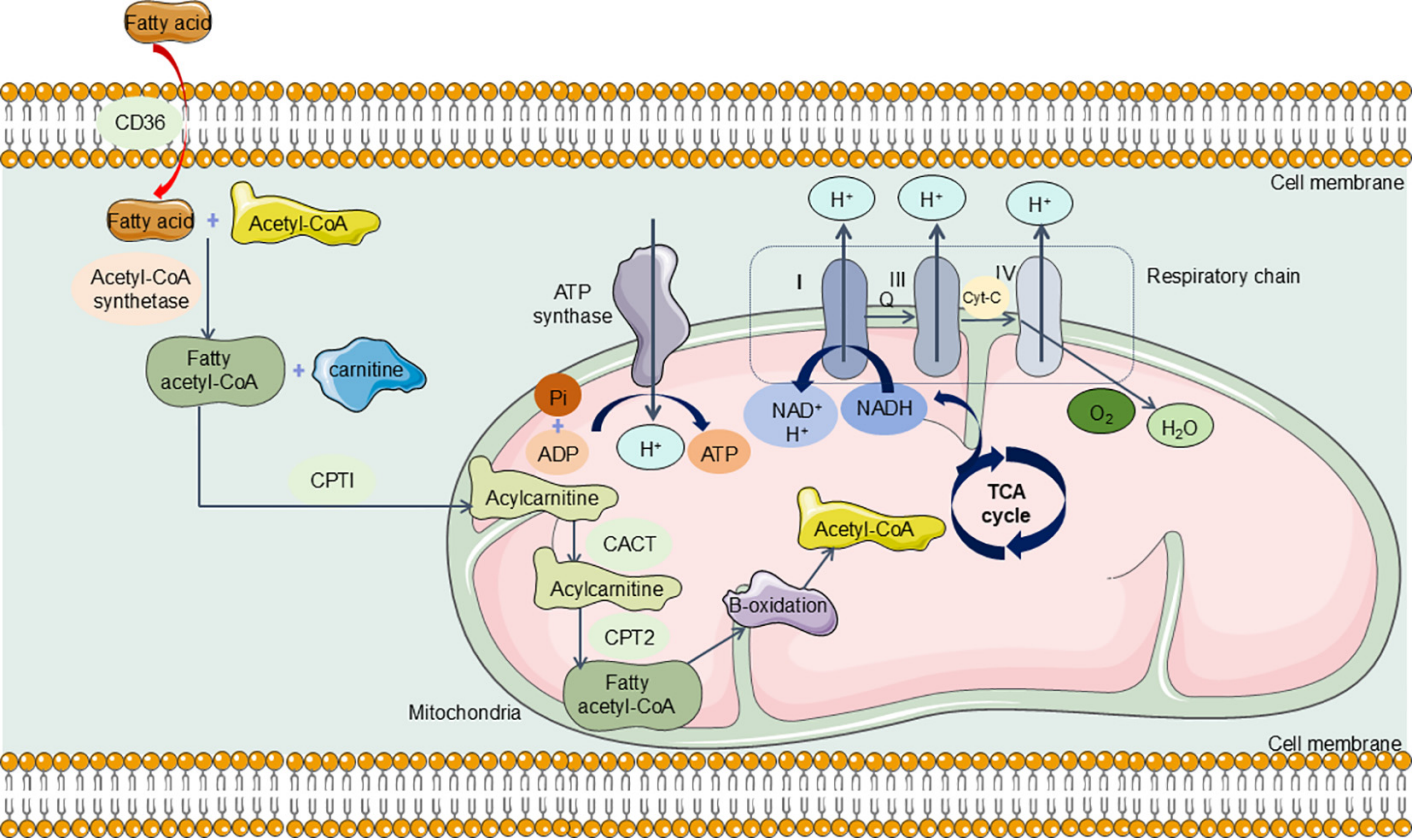
The Normal Metabolism in Renal TECs. TECs can absorb long-chain fatty acids via transport proteins, which are then oxidized within the mitochondria. This oxidative process yields acetyl-CoA and reduced nicotinamide adenine dinucleotide (NADH). Acetyl-CoA enters the tricarboxylic acid (TCA) cycle, producing additional NADH, which subsequently drive the electron transport chain to generate ATP. CD36, CPTI, carnitine palmitoyl transferase I; CACT, carnitine-acylcarnitine translocase; CPT2, carnitine palmitoyl transferase 2; TCA cycle, tricarboxylic acid cycle; NADH, Nicotinamide adenine dinucleotide.

**Figure 6:**
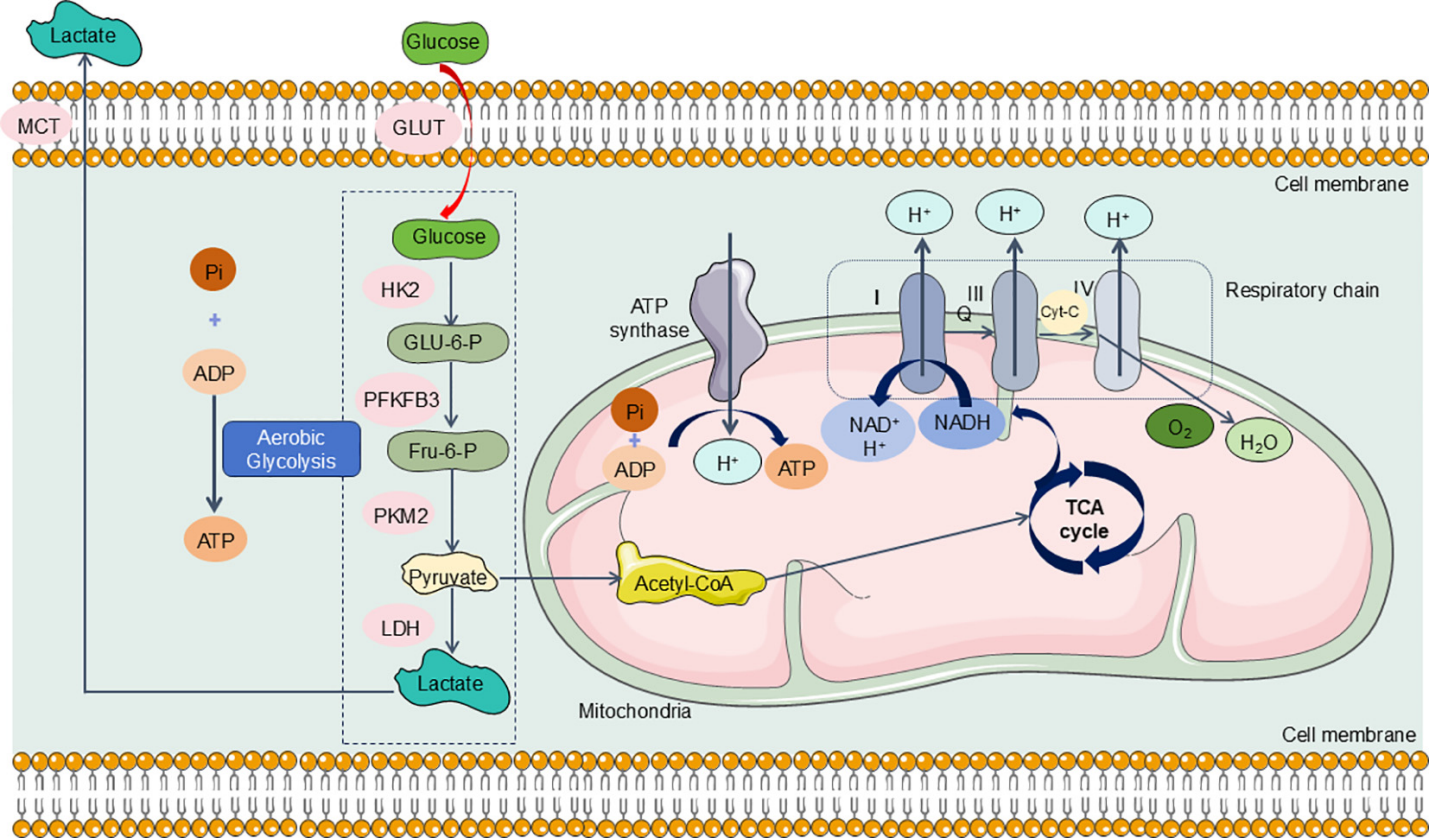
TECs experience a metabolic transition from FAO-dependent OXPHOS to a predominantly glycolytic metabolism in SA-AKI. During the progression of SA-AKI, the metabolism of TECs has changed from OXPHOS to aerobic glycolysis, specifically, glucose undergoes a series of enzymatic reactions to be metabolized into pyruvate and generates ATP in the cytoplasm of TECs. Under hypoxic conditions, pyruvate cannot enter the mitochondria for aerobic respiration and is instead reduced to lactate via action of LDH. GLUT, glucose transporter; HK2, hexokinase2; PFKFB3, 6-phosphofructo-2-kinase/fructose-2,6-biphosphatase 3; PKM2, Pyruvate kinase M2; MCT, Medium chain triglyceride; LDH, lactate dehydrogenase.

As previously outlined, the kidneys rank second among the body’s organs in mitochondrial density. Mitochondria in the kidneys generate ATP crucial for powering the Na+-K+ ATPase pump, which is integral in establishing the ion gradients necessary for its function. Research indicates that the energy expenditure associated with this reabsorption process constitutes approximately 70% of the total renal energy expenditure (161). To fulfill these high energy demands, renal tubular epithelial cells (TECs) predominantly utilize FAO and OXPHOS for effective energy production (162, 163). Specifically, TECs absorb long-chain fatty acids via transport proteins, which are then oxidized within the mitochondria (164). This oxidative process yields acetyl-CoA, reduced flavin adenine dinucleotide (FADH2), and reduced nicotinamide adenine dinucleotide (NADH) (165). Acetyl-CoA enters the tricarboxylic acid (TCA) cycle, producing additional FADH2 and NADH, which subsequently drive the electron transport chain to generate ATP (166) (**Figure 5**). In the aftermath of acute kidney injury induced by sepsis, renal TECs experience a metabolic transition from FAO-dependent OXPHOS to a predominantly glycolytic metabolism. During glycolysis, a significant amount of pyruvate is generated but diverted from mitochondrial entry and is instead converted to lactate through the action of lactate dehydrogenase (LDH). This metabolic adaptation is essential for enhancing ATP production via glycolysis, thereby addressing the increased energy demands imposed by sepsis and SA-AKI (**Figure 6**).

In the early metabolic response to SA-AKI, renal tubular epithelial cells, akin to immune cells, enter a pro-inflammatory phase characterized by the activation of aerobic glycolysis via the Akt/mTORC1/Hypoxia-Inducible Factor (HIF)-1α signaling pathway. HIF-1α promotes the conversion of pyruvate to lactate and, in synergy with pyruvate dehydrogenase kinase (PDHK), inhibits the transformation of lactate to acetyl-CoA (167, 168). This inhibition disrupts the Krebs cycle, thereby diminishing OXPHOS and its associated metabolic processes (167, 168). Li et al. established a sepsis mouse model via CLP, revealing a significant reduction in renal fatty acid oxidase expression and a pronounced increase in glycolytic enzyme expression (169). Furthermore, subsequent studies indicated that 8 hours after CLP, notable metabolic alterations occurred in renal TECs, characterized by elevated levels of glycolytic intermediates and decreased tricarboxylic acid (TCA) cycle intermediates (170). In parallel, glycolytic reprogramming has been identified as a crucial mechanism for establishing trained immunity during the early stages of LPS-induced acute kidney injury (143). In this LPS-induced SA-AKI model, hexokinase (HK) activity was significantly elevated, facilitating the activation of the pentose phosphate pathway (PPP), which is essential for glutathione (GSH) maintenance (143). Thus, it is plausible that under septic conditions, renal tubular epithelial cells adapt their metabolism through analogous mechanisms to respond to alterations in other cell types (171). This metabolic reconfiguration represents a protective adaptive response aimed at enhancing infection tolerance, mitigating cellular damage, and preserving cell viability (171).

To sum it up, during the onset of sepsis, the body’s metabolism shifts towards glycolysis, which may enhance tolerance and promote cell survival. Nevertheless, prolonged reliance on this metabolic pathway can ultimately be detrimental. A transition back to OXPHOS is crucial for maintaining cellular function. This metabolic shift underscores the rationale for targeting metabolic reprogramming as a therapeutic approach for SA-AKI. Post-SA-AKI onset, metabolic reprogramming in renal TECs results in decreased utilization of free fatty acids and subsequent lipid accumulation (172). Lipid accumulation might play an indispensable part in the advancement of renal tubular fibrosis and glomerulosclerosis (173). Recent studies have demonstrated that the activation of uncoupling protein 1 (UCP1) can mitigate lipid accumulation, thereby attenuating the progression of acute kidney injury via the AMPK/ULK1/autophagy pathway (174). Additionally, there is compelling evidence suggesting that the prompt re-establishment of OXPHOS and FAO can substantially salvage renal tubular ion transport and preserve kidney function (175).

In this review, we primarily explore the impact of metabolic reprogramming of renal cells during sepsis on kidney injury, as well as how this reprogramming can lead to immune dysregulation and subsequently induce mitochondrial damage. Overall, we posit that moderate metabolic reprogramming, in response to various inflammatory stressors, may help delay the progression of kidney damage. However, excessive metabolic reprogramming can result in decreased utilization of free fatty acids, leading to lipid accumulation that can harm renal tubular and glomerular structures, thereby accelerating sepsis-related kidney injury. Unfortunately, current research on the specific molecular mechanisms by which metabolic reprogramming induces kidney injury remains insufficient. Thus, our aim is to lay a foundation for future studies to delve deeper into the specific roles and biological significance of metabolic reprogramming in SA-AKI.

## 4 Targeted therapies for SA-AKI

As outlined, aberrant inflammatory responses and altered metabolic pathways are central to the development of SA-AKI. Hence, targeting regulated cell death pathways linked to inflammation and metabolic reprogramming mediated by mitochondria is a key strategy for managing SA-AKI (**Table 1**). For instance, Andrographolide has been demonstrated to mitigate iron accumulation and lipid peroxidation via the Nrf2/FSP1 axis, enhance SLC7A11 and GPX4 expression, and prevent ferroptosis in HK-2 renal tubular epithelial cells, thus attenuated septic kidney injury (115). Furthermore, celastrol and shikonin have been identified as inhibitors of the glycolytic enzyme PKM2, which helps alleviate renal damage in SA-AKI (210, 211). Since both regulatory cell death and metabolic reprogramming driven by inflammatory dysregulation are implicated in sepsis and associated renal injury, exploring multi-targeted therapeutic approaches for SA-AKI represents a significant research frontier.

**Table 1:** Therapeutic Strategies Based on RCD associated with inflammatory response and metabolic reprogramming in SA-AKI.

Mechanism	Therapy	Target/ signaling pathway	Effect for Target/ signaling pathway	In-vivo model	In-vivo subject	In-vitro model	In-vitro subject	Effect	Reference
Pyroptosis	Wild-Type p53-Induced Phosphatase 1 (WIP1/PPM1D)	p38 MAPK signaling	Downregulation	LPS	mice	LPS	HK2 cell	Inhibition	(176)
	Zn2+	NLRP3 inflammasome	Downregulation	CLP	rat	LPS	HK2 cell	Inhibition	(177)
	chlorogenic acid (CGA)	NLRP3 inflammasome	Downregulation	CLP	rat	–	–	Inhibition	(178)
	tissue inhibitor of metalloproteinases 2 (TIMP2)	Caspase-1、NLRP3、GSDMD	Downregulation	CLP	rat	LPS	HK2 cell	Inhibition	(21)
	Maresin-1	Caspase-1、NLRP3、GSDMD	Downregulation	CLP	rat	–	–	Inhibition	(179)
	Alamandine (ALA)	Caspase-1	Downregulation	LPS	rat	–	–	Inhibition	(180)
	P. cuspidatum extracts (PCE)	NF-κB	Downregulation	LPS	rat	LPS	HK2 cell	Inhibition	(181)
	Immune Response Gene-1 [IRG1]/itaconate	GSDMD	Downregulation	LPS	mice	–	–	Inhibition	(29)
	Erbin	NLRP3 inflammasome	Downregulation	CLP	rat	LPS	HK2 cell	Inhibition	(182)
	Theaflavin	NLRP3 inflammasome	Downregulation	LPS	mice	LPS	macrophages	Inhibition	(183)
	saroglitazar (SAR)	Caspase-11、GSDMD	Downregulation	LPS	rat	–	–	Inhibition	(184)
	Protein Kinase R Inhibitor C16	apoptosis-associated speck-like protein、NACHT, LRR, NLR Family Pyrin Domain-Containing 3; caspase-1	Downregulation	LPS	mice	–	–	Inhibition	(185)
	Mdivi-1	NLRP3 inflammasome	Downregulation	LPS	mice	LPS	HK2 cell	Inhibition	(186)
	Thymoquinone	NLRP3、caspase-1、caspase-3	Downregulation	CLP	BALB/c mice	–	–	Inhibition	(187)
	Micheliolide	NLRP3 inflammasome	Downregulation	LPS	mice	LPS	HK2 cell	Inhibition	(188)
Necroptosis	Dexmedetomidine	EMT	Downregulation	LPS	mice	LPS	HK2 cell	Inhibition	(57)
	Gut-derived 4-hydroxyphenylacetic acid	apoptosis repressor with caspase recruitment domain (ARC)	Upregulation	CLP	mice	LPS	HK2 cell	Inhibition	(16)
	Compound 4-155	RIPK1, RIPK3, MLKL	Downregulation	TNF	mice	–	–	Inhibition	(189)
Autophagy	Resveratrol	beclin1	Upregulation	CLP	mice	–	–	Upregulation	(80)
	Micheliolide	Nrf2/PINK1/Parkin	Upregulation	LPS	mice	LPS	HK2 cell	Upregulation	(188)
	Zn2+	SIRT7	Upregulation	CLP	rat	LPS	HK2 cell	Upregulation	(177)
	Rapamycin	Beclin-1 levels, LC3II/LC3I ratio	Upregulation	CLP	rat	–	–	Upregulation	(190)
	FTO	SNHG14/miR-373-3p/ATG7	Downregulation	–	–	LPS	mice	Inhibition	(191)
	Alcohol dehydrogenase 1	PINK1-Parkin	Upregulation	LPS	mice	–	–	Upregulation	(192)
	Liensinine	JNK/ p38-ATF 2 axis	Upregulation	LPS	mice	LPS	HK2 cell	Inhibition	(193)
	Ulinastatin	LC3II	Downregulation	CLP	rat	–	–	Inhibition	(194)
	Ascorbate	SVCT-1 and -2	Upregulation	LPS	mice	LPS	HK2 cell	Upregulation	(195)
	procyanidin B2	Nrf2 nuclear translocation	Upregulation	LPS	mice	–	–	Upregulation	(196)
	Dexmedetomidine	α2-AR/AMPK/mTOR	Upregulation	LPS	rat	–	–	Upregulation	(197)
Ferroptosis	Andrographolide	SLC7A11, GPX4	Upregulation	CLP	rat	LPS	HK2 cell	Inhibition	(115)
	Klotho	Nrf2	Upregulation	CLP	rat	LPS	HK2 cell	Inhibition	(198)
	Melittin	GPX4	Upregulation	LPS	mice	LPS	HK2 cell	Inhibition	(199)
	Dexmedetomidine	GPX4	Upregulation	CLP	mice	LPS	HK2 cell	Inhibition	(200)
	GYY4137	GPX4	Upregulation	CLP	mice	LPS	MRGECs	Inhibition	(201)
	Melatonin	Nrf2/HO-1 signaling pathway	Upregulation	CLP, LPS	mice	–	–	Inhibition	(202)
	Ginsenoside Rg1	GPX4, FSP1, GSH	Upregulation	CLP	mice	LPS	HK2 cell	Inhibition	(203)
	Irisin	SIRT1/Nrf2	Upregulation	CLP	mice	LPS	HK2 cell	Inhibition	(111)
	Maresin conjugates in tissue regeneration 1 (MCTR1	Nrf2	Upregulation	CLP	mice	–	–	Inhibition	(204)
Metabolism reprogramming	2−deoxy−D−glucose (2−DG)	lactate/Sirtuin 3/AMPK	Upregulation	–	–	LPS	HK2 cell	Inhibition	(205)
	uncoupling protein-2 (UCP2)	Warburg	Downregulation	CLP	mice	LPS	HK2 cell	Inhibition	(206)
	Retinoic acid receptor (RAR)	Kim-1-dependent Efferocytosis	Upregulation	LPS	mice	–	–	Inhibition	(207)
	Vitamin D- VDR (vitamin D receptor)	lactate	Downregulation	LPS	mice	LPS	HK2 cell	Inhibition	(208)
	recombinant human erythropoietin (rhEPO)	PGC-1α	Upregulation	CLP	mice	LPS	HK2 cell, TEC cell	Inhibition	(209)
	Peroxisome proliferator-activated receptor-α (PPAR-α)	FAO enzyme	Upregulation	LPS	mice	–	–	Inhibition	(160)
	shikonin	PKM2	Downregulation	LPS	mice	–	–	Inhibition	(210)
	Celastrol	PKM2	Downregulation	LPS	mice	LPS	macrophage	Inhibition	(211)

## 5 Conclusion

In general, we examine the pathogenesis and therapeutic approaches for SA-AKI, focusing on the roles of dysregulated inflammation and metabolic reprogramming. Evidence suggests that, in the early stages of SA-AKI, well-regulated inflammatory responses and metabolic reprogramming may confer renal protection by activating adaptive mechanisms. However, prolonged dependence on aerobic glycolysis and dysregulated inflammation might be associated with detrimental effects, including tubular atrophy and fibrosis. Presently, several biomolecules and phytochemicals exhibit promising potential in the management of SA-AKI, yet additional clinical trials are needed to establish their safety and efficacy. Furthermore, to mitigate the high mortality rates associated with SA-AKI, it is imperative to conduct in-depth studies on the signaling mechanisms involved, identify viable therapeutic targets, and develop novel and effective pharmacological treatments.

## References

[1] Stephens AJ Chauhan SP Barton JR Sibai BM. Maternal sepsis: A review of national and international guidelines. Am J Perinatol. (2023) 40:718–30. doi: 10.1055/s-0041-1736382 34634831

[2] Zhou J Tian H Du X Xi X An Y Duan M. Population-based epidemiology of sepsis in a subdistrict of beijing. Crit Care Med. (2017) 45:1168–76. doi: 10.1097/CCM.0000000000002414 28422777

[3] Cheng B Li Z Wang J Xie G Liu X Xu Z. Comparison of the performance between sepsis-1 and sepsis-3 in ICUs in China: A retrospective multicenter study. Shock. (2017) 48:301–6. doi: 10.1097/SHK.0000000000000868 PMC5516667 28448400

[4] Lakshmikanth CL Jacob SP Chaithra VH De Castro-Faria-Neto HC Marathe GK. Sepsis: in search of cure. Inflamm Res. (2016) 65:587–602. doi: 10.1007/s00011-016-0937-y 26995266

[5] Mayr FB Prescott HC. Identifying survivors of sepsis at risk for adverse cardiovascular outcomes. Am J Respir Crit Care Med. (2021) 204:500–1. doi: 10.1164/rccm.202105-1322ED PMC8491261 34139144

[6] Eisen DP Leder K Woods RL Lockery JE McGuinness SL Wolfe R. Effect of aspirin on deaths associated with sepsis in healthy older people (ANTISEPSIS): a randomised, double-blind, placebo-controlled primary prevention trial. Lancet Respir Med. (2021) 9:186–95. doi: 10.1016/S2213-2600(20)30411-2 PMC7957956 32950072

[7] Liu Q Song H Andersson TML Magnusson PKE Zhu J Smedby KE. Psychiatric disorders are associated with increased risk of sepsis following a cancer diagnosis. Cancer Res. (2020) 80:3436–42. doi: 10.1158/0008-5472.CAN-20-0502 32532824

[8] Peerapornratana S Manrique-Caballero CL Gómez H Kellum JA. Acute kidney injury from sepsis: current concepts, epidemiology, pathophysiology, prevention and treatment. Kidney Int. (2019) 96:1083–99. doi: 10.1016/j.kint.2019.05.026 PMC6920048 31443997

[9] Bellomo R Kellum JA Ronco C Wald R Martensson J Maiden M. Acute kidney injury in sepsis. Intensive Care Med. (2017) 43:816–28. doi: 10.1007/s00134-017-4755-7 28364303

[10] He F-F Wang YM Chen YY Huang W Li ZQ Zhang C. Sepsis-induced AKI: From pathogenesis to therapeutic approaches. Front Pharmacol. (2022) 13:981578. doi: 10.3389/fphar.2022.981578 36188562 PMC9522319

[11] Opal SM van der Poll T. Endothelial barrier dysfunction in septic shock. J Intern Med. (2015) 277:277–93. doi: 10.1111/joim.2015.277.issue-3 25418337

[12] van der Poll T Shankar-Hari M Wiersinga WJ. The immunology of sepsis. Immunity. (2021) 54:2450–64. doi: 10.1016/j.immuni.2021.10.012 34758337

[13] Tang D Kang R Berghe TV Vandenabeele P Kroemer G. The molecular machinery of regulated cell death. Cell Res. (2019) 29:347–64. doi: 10.1038/s41422-019-0164-5 PMC6796845 30948788

[14] Bayır H Dixon SJ Tyurina YY Kellum JA Kagan VE. Ferroptotic mechanisms and therapeutic targeting of iron metabolism and lipid peroxidation in the kidney. Nat Rev Nephrol. (2023) 19:315–36. doi: 10.1038/s41581-023-00689-x 36922653

[15] Li Y Zhang J Zhai P Hu C Suo J Wang J. The potential biomarker TIFA regulates pyroptosis in sepsis-induced acute kidney injury. Int Immunopharmacol. (2023) 115:109580. doi: 10.1016/j.intimp.2022.109580 36586274

[16] An S Yao Y Wu J Hu H Wu J Sun M. Gut-derived 4-hydroxyphenylacetic acid attenuates sepsis-induced acute kidney injury by upregulating ARC to inhibit necroptosis. Biochim Biophys Acta Mol Basis Dis. (2024) 1870:166876. doi: 10.1016/j.bbadis.2023.166876 37714058

[17] Sun M Li J Mao L Wu J Deng Z He M. p53 deacetylation alleviates sepsis-induced acute kidney injury by promoting autophagy. Front Immunol. (2021) 12:685523. doi: 10.3389/fimmu.2021.685523 34335587 PMC8318785

[18] Ding J Wang K Liu W She Y Sun Q Shi J. Pore-forming activity and structural autoinhibition of the gasdermin family. Nature. (2016) 535:111–6. doi: 10.1038/nature18590 27281216

[19] Miao EA Leaf IA Treuting PM Mao DP Dors M Sarkar A. Caspase-1-induced pyroptosis is an innate immune effector mechanism against intracellular bacteria. Nat Immunol. (2010) 11:1136–42. doi: 10.1038/ni.1960 PMC3058225 21057511

[20] Li T Sun H Li Y Su L Jiang J Liu Y. Downregulation of macrophage migration inhibitory factor attenuates NLRP3 inflammasome mediated pyroptosis in sepsis-induced AKI. Cell Death Discovery. (2022) 8:61. doi: 10.1038/s41420-022-00859-z 35165294 PMC8844278

[21] Xu D Jiang J Liu Y Pang J Suo J Li Y. TIMP2 protects against sepsis-associated acute kidney injury by cAMP/NLRP3 axis-mediated pyroptosis. Am J Physiol Cell Physiol 326 C1353–C1366. (2024) 326:C1353–66. doi: 10.1152/ajpcell.00577.2023 38497110

[22] Liu X Lieberman J. Inflammasome-independent pyroptosis. Curr Opin Immunol. (2024) 88:102432. doi: 10.1016/j.coi.2024.102432 38875738

[23] Zhu C Xu S Jiang R Yu Y Bian J Zou Z. The gasdermin family: emerging therapeutic targets in diseases. Signal Transduct Target Ther. (2024) 9:87. doi: 10.1038/s41392-024-01801-8 38584157 PMC10999458

[24] Ai Y Meng Y Yan B Zhou Q Wang X. The biochemical pathways of apoptotic, necroptotic, pyroptotic, and ferroptotic cell death. Mol Cell. (2024) 84:170–9. doi: 10.1016/j.molcel.2023.11.040 38181758

[25] Atabaki R Khaleghzadeh-Ahangar H Esmaeili N Mohseni-Moghaddam P. Role of pyroptosis, a pro-inflammatory programmed cell death, in epilepsy. Cell Mol Neurobiol. (2023) 43:1049–59. doi: 10.1007/s10571-022-01250-3 PMC11414441 35835968

[26] Liu X Zhang Z Ruan J Pan Y Magupalli VG Wu H. Inflammasome-activated gasdermin D causes pyroptosis by forming membrane pores. Nature. (2016) 535:153–8. doi: 10.1038/nature18629 PMC5539988 27383986

[27] Barnett KC Ting JP-Y. Mitochondrial GSDMD pores DAMPen pyroptosis. Immunity. (2020) 52:424–6. doi: 10.1016/j.immuni.2020.02.012 PMC7337261 32187511

[28] Xiaodong L Xuejun X. GSDMD-mediated pyroptosis in retinal vascular inflammatory diseases: a review. Int Ophthalmol. (2023) 43:1405–11. doi: 10.1007/s10792-022-02506-z 36068399

[29] Yang W Wang Y Huang Y Wang T Li C Zhang P. Immune Response Gene-1 [IRG1]/itaconate protect against multi-organ injury via inhibiting gasdermin D-mediated pyroptosis and inflammatory response. Inflammopharmacology. (2024) 32:419–32. doi: 10.1007/s10787-023-01278-x 37470905

[30] Islamuddin M Qin X. Renal macrophages and NLRP3 inflammasomes in kidney diseases and therapeutics. Cell Death Discovery. (2024) 10:229. doi: 10.1038/s41420-024-01996-3 38740765 PMC11091222

[31] Yao J Sterling K Wang Z Zhang Y Song W. The role of inflammasomes in human diseases and their potential as therapeutic targets. Signal Transduct Target Ther. (2024) 9:10. doi: 10.1038/s41392-023-01687-y 38177104 PMC10766654

[32] Dolinay T Kim YS Howrylak J Hunninghake GM An CH Fredenburgh L. Inflammasome-regulated cytokines are critical mediators of acute lung injury. Am J Respir Crit Care Med. (2012) 185:1225–34. doi: 10.1164/rccm.201201-0003OC PMC3373064 22461369

[33] Homsy E Das S Consiglio P McAtee C Zachman A Nagaraja H. Circulating gasdermin-D in critically ill patients. Crit Care Explor. (2019) 1:e0039. doi: 10.1097/CCE.0000000000000039 32166281 PMC7063936

[34] Huang W Wang X Xie F Zhang H Liu D. Serum NLRP3: A biomarker for identifying high-risk septic patients. Cytokine. (2022) 149:155725. doi: 10.1016/j.cyto.2021.155725 34634653

[35] Huang G Bao J Shao X Zhou W Wu B Ni Z. Inhibiting pannexin-1 alleviates sepsis-induced acute kidney injury via decreasing NLRP3 inflammasome activation and cell apoptosis. Life Sci. (2020) 254:117791. doi: 10.1016/j.lfs.2020.117791 32416166

[36] Gotts JE Matthay MA. Sepsis: pathophysiology and clinical management. BMJ (2016) 353:i1585. doi: 10.1136/bmj.i1585 27217054

[37] Zafrani L Payen D Azoulay E Ince C. The microcirculation of the septic kidney. Semin Nephrol. (2015) 35:75–84. doi: 10.1016/j.semnephrol.2015.01.008 25795501

[38] Wu L Tiwari MM Messer KJ Holthoff JH Gokden N Brock RW. Peritubular capillary dysfunction and renal tubular epithelial cell stress following lipopolysaccharide administration in mice. Am J Physiol Renal Physiol. (2007) 292:F261–8. doi: 10.1152/ajprenal.00263.2006 16926442

[39] Bertheloot D Latz E Franklin BS. Necroptosis, pyroptosis and apoptosis: an intricate game of cell death. Cell Mol Immunol. (2021) 18:1106–21. doi: 10.1038/s41423-020-00630-3 PMC8008022 33785842

[40] Khoury MK Gupta K Franco SR Liu B. Necroptosis in the pathophysiology of disease. Am J Pathol. (2020) 190:272–85. doi: 10.1016/j.ajpath.2019.10.012 PMC6983729 31783008

[41] Newton K Strasser A Kayagaki N Dixit VM. Cell death. Cell. (2024) 187:235–56. doi: 10.1016/j.cell.2023.11.044 38242081

[42] Han J Zhong C-Q Zhang D-W. Programmed necrosis: backup to and competitor with apoptosis in the immune system. Nat Immunol. (2011) 12:1143–9. doi: 10.1038/ni.2159 22089220

[43] Moreno-Gonzalez G Vandenabeele P Krysko DV. Necroptosis: A novel cell death modality and its potential relevance for critical care medicine. Am J Respir Crit Care Med. (2016) 194:415–28. doi: 10.1164/rccm.201510-2106CI 27285640

[44] Delano MJ Ward PA. The immune system’s role in sepsis progression, resolution, and long-term outcome. Immunol Rev. (2016) 274:330–53. doi: 10.1111/imr.2016.274.issue-1 PMC5111634 27782333

[45] Ehrman RR Sullivan AN Favot MJ Sherwin RL Reynolds CA Abidov A. Pathophysiology, echocardiographic evaluation, biomarker findings, and prognostic implications of septic cardiomyopathy: a review of the literature. Crit Care. (2018) 22:112. doi: 10.1186/s13054-018-2043-8 29724231 PMC5934857

[46] Laster SM Wood JG Gooding LR. Tumor necrosis factor can induce both apoptic and necrotic forms of cell lysis. J Immunol. (1988) 141:2629–34. doi: 10.4049/jimmunol.141.8.2629 3171180

[47] Holler N Zaru R Micheau O Thome M Attinger A Valitutti S. Fas triggers an alternative, caspase-8-independent cell death pathway using the kinase RIP as effector molecule. Nat Immunol. (2000) 1:489–95. doi: 10.1038/82732 11101870

[48] He S Liang Y Shao F Wang X. Toll-like receptors activate programmed necrosis in macrophages through a receptor-interacting kinase-3-mediated pathway. Proc Natl Acad Sci U.S.A. (2011) 108:20054–9. doi: 10.1073/pnas.1116302108 PMC3250173 22123964

[49] Tenev T Bianchi K Darding M Broemer M Langlais C Wallberg F. The Ripoptosome, a signaling platform that assembles in response to genotoxic stress and loss of IAPs. Mol Cell. (2011) 43:432–48. doi: 10.1016/j.molcel.2011.06.006 21737329

[50] Feoktistova M Geserick P Kellert B Dimitrova DP Langlais C Hupe M. cIAPs block Ripoptosome formation, a RIP1/caspase-8 containing intracellular cell death complex differentially regulated by cFLIP isoforms. Mol Cell. (2011) 43:449–63. doi: 10.1016/j.molcel.2011.06.011 PMC3163271 21737330

[51] Martin EL Ranieri VM. Phosphorylation mechanisms in intensive care medicine. Intensive Care Med. (2011) 37:7–18. doi: 10.1007/s00134-010-2023-1 20820992

[52] Li J McQuade T Siemer AB Napetschnig J Moriwaki K Hsiao YS. The RIP1/RIP3 necrosome forms a functional amyloid signaling complex required for programmed necrosis. Cell. (2012) 150:339–50. doi: 10.1016/j.cell.2012.06.019 PMC3664196 22817896

[53] Zhao J Jitkaew S Cai Z Choksi S Li Q Luo J. Mixed lineage kinase domain-like is a key receptor interacting protein 3 downstream component of TNF-induced necrosis. Proc Natl Acad Sci U.S.A. (2012) 109:5322–7. doi: 10.1073/pnas.1200012109 PMC3325682 22421439

[54] Murphy JM Czabotar PE Hildebrand JM Lucet IS Zhang JG Alvarez-Diaz S. The pseudokinase MLKL mediates necroptosis via a molecular switch mechanism. Immunity. (2013) 39:443–53. doi: 10.1016/j.immuni.2013.06.018 24012422

[55] Upton JW Kaiser WJ Mocarski ES. DAI/ZBP1/DLM-1 complexes with RIP3 to mediate virus-induced programmed necrosis that is targeted by murine cytomegalovirus vIRA. Cell Host Microbe. (2012) 11:290–7. doi: 10.1016/j.chom.2012.01.016 PMC3531981 22423968

[56] Maelfait J Liverpool L Bridgeman A Ragan KB Upton JW Rehwinkel J. Sensing of viral and endogenous RNA by ZBP1/DAI induces necroptosis. EMBO J. (2017) 36:2529–43. doi: 10.15252/embj.201796476 PMC5579359 28716805

[57] Sun Q Kamath P Sun Y Liang M Wu L Chang E. Dexmedetomidine attenuates lipopolysaccharide-induced renal cell fibrotic phenotypic changes by inhibiting necroinflammation via activating α2-adrenoceptor: A combined randomised animal and. Vitro study. BioMed Pharmacother. (2024) 174:116462. doi: 10.1016/j.biopha.2024.116462 38513598

[58] Wang B Li J Gao HM Xing YH Lin Z Li HJ. Necroptosis regulated proteins expression is an early prognostic biomarker in patient with sepsis: a prospective observational study. Oncotarget. (2017) 8:84066–73. doi: 10.18632/oncotarget.21099 PMC5663577 29137405

[59] Mallarpu CS Ponnana M Prasad S Singarapu M Kim J Haririparsa N. Distinct cell death markers identified in critical care patient survivors diagnosed with sepsis. Immunol Lett. (2021) 231:1–10. doi: 10.1016/j.imlet.2020.12.009 33406390

[60] Schenck EJ Ma KC Price DR Nicholson T Oromendia C Gentzler ER. Circulating cell death biomarker TRAIL is associated with increased organ dysfunction in sepsis. JCI Insight. (2019) 4:e127143, 127143. doi: 10.1172/jci.insight.127143 31045578 PMC6538332

[61] Rosenbaum DM Degterev A David J Rosenbaum PS Roth S Grotta JC. Necroptosis, a novel form of caspase-independent cell death, contributes to neuronal damage in a retinal ischemia-reperfusion injury model. J Neurosci Res. (2010) 88:1569–76. doi: 10.1002/jnr.22314 PMC7713513 20025059

[62] Kaczmarek A Vandenabeele P Krysko DV. Necroptosis: the release of damage-associated molecular patterns and its physiological relevance. Immunity. (2013) 38:209–23. doi: 10.1016/j.immuni.2013.02.003 23438821

[63] Newton K Dugger DL Maltzman A Greve JM Hedehus M Martin-McNulty B. RIPK3 deficiency or catalytically inactive RIPK1 provides greater benefit than MLKL deficiency in mouse models of inflammation and tissue injury. Cell Death Differ. (2016) 23:1565–76. doi: 10.1038/cdd.2016.46 PMC5072432 27177019

[64] Rathkey JK Zhao J Liu Z Chen Y Yang J Kondolf HC. Chemical disruption of the pyroptotic pore-forming protein gasdermin D inhibits inflammatory cell death and sepsis. Sci Immunol. (2018) 3:eaat2738. doi: 10.1126/sciimmunol.aat2738 30143556 PMC6462819

[65] Bland RD Clarke TL Harden LB. Rapid infusion of sodium bicarbonate and albumin into high-risk premature infants soon after birth: a controlled, prospective trial. Am J Obstet Gynecol. (1976) 124:263–7. doi: 10.1016/0002-9378(76)90154-X 2013

[66] Kitur K Wachtel S Brown A Wickersham M Paulino F Peñaloza HF. Necroptosis promotes staphylococcus aureus clearance by inhibiting excessive inflammatory signaling. Cell Rep. (2016) 16:2219–30. doi: 10.1016/j.celrep.2016.07.039 PMC5001919 27524612

[67] Esrefoglu M. Harnessing autophagy: A potential breakthrough in digestive disease treatment. World J Gastroenterol. (2024) 30:3036–43. doi: 10.3748/wjg.v30.i24.3036 PMC11230060 38983959

[68] Choi ME. Autophagy in kidney disease. Annu Rev Physiol. (2020) 82:297–322. doi: 10.1146/annurev-physiol-021119-034658 31640469

[69] Choi AMK Ryter SW Levine B. Autophagy in human health and disease. N Engl J Med. (2013) 368:651–62. doi: 10.1056/NEJMra1205406 23406030

[70] Ho J Yu J Wong SH Zhang L Liu X Wong WT. Autophagy in sepsis: Degradation into exhaustion? Autophagy. (2016) 12:1073–82. doi: 10.1080/15548627.2016.1179410 PMC4990998 27172163

[71] Hsiao H-W Tsai KL Wang LF Chen YH Chiang PC Chuang SM. The decline of autophagy contributes to proximal tubular dysfunction during sepsis. Shock. (2012) 37:289–96. doi: 10.1097/SHK.0b013e318240b52a 22089196

[72] Yin X Xin H Mao S Wu G Guo L. The role of autophagy in sepsis: protection and injury to organs. Front Physiol. (2019) 10:1071. doi: 10.3389/fphys.2019.01071 31507440 PMC6716215

[73] Leventhal JS Ni J Osmond M Lee K Gusella GL Salem F. Autophagy limits endotoxemic acute kidney injury and alters renal tubular epithelial cell cytokine expression. PloS One. (2016) 11:e0150001. doi: 10.1371/journal.pone.0150001 26990086 PMC4798771

[74] Wu Y Wang L Meng L Cao GK Zhao YL Zhang Y. Biological effects of autophagy in mice with sepsis-induced acute kidney injury. Exp Ther Med. (2019) 17:316–22. doi: 10.3892/etm.2018.6899 PMC6307358 30651797

[75] Bhatia D Choi ME. Autophagy in kidney disease: Advances and therapeutic potential. Prog Mol Biol Transl Sci. (2020) 172:107–33. doi: 10.1016/bs.pmbts.2020.01.008 32620239

[76] Liu M Chen X. Human umbilical cord-derived mesenchymal stem cells-exosomes-delivered miR-375 targets HDAC4 to promote autophagy and suppress T cell apoptosis in sepsis-associated acute kidney injury. Appl Biochem Biotechnol. (2024). doi: 10.1007/s12010-024-04963-x 38668845

[77] Li K Liu TX Li JF Ma YR Liu ML Wang YQ. rhEPO inhibited cell apoptosis to alleviate acute kidney injury in sepsis by AMPK/SIRT1 activated autophagy. Biochem Biophys Res Commun. (2019) 517:557–65. doi: 10.1016/j.bbrc.2019.07.027 31383361

[78] Hsieh C-H Pai P-Y Hsueh H-W Yuan S-S Hsieh Y-C. Complete induction of autophagy is essential for cardioprotection in sepsis. Ann Surg. (2011) 253:1190–200. doi: 10.1097/SLA.0b013e318214b67e 21412148

[79] Bai X Zhu Y Jie J Li D Song L Luo J. Maackiain protects against sepsis via activating AMPK/Nrf2/HO-1 pathway. Int Immunopharmacol. (2022) 108:108710. doi: 10.1016/j.intimp.2022.108710 35405595

[80] Deng Z Sun M Wu J Fang H Cai S An S. SIRT1 attenuates sepsis-induced acute kidney injury via Beclin1 deacetylation-mediated autophagy activation. Cell Death Dis. (2021) 12:217. doi: 10.1038/s41419-021-03508-y 33637691 PMC7910451

[81] Zhao W Zhang L Chen R Lu H Sui M Zhu Y. SIRT3 protects against acute kidney injury via AMPK/mTOR-regulated autophagy. Front Physiol. (2018) 9:1526. doi: 10.3389/fphys.2018.01526 30487750 PMC6246697

[82] Deng Z He M Hu H Zhang W Zhang Y Ge Y. Melatonin attenuates sepsis-induced acute kidney injury by promoting mitophagy through SIRT3-mediated TFAM deacetylation. Autophagy. (2024) 20:151–65. doi: 10.1080/15548627.2023.2252265 PMC10761103 37651673

[83] Zhang Y Wang L Meng L Cao G Wu Y. Sirtuin 6 overexpression relieves sepsis-induced acute kidney injury by promoting autophagy. Cell Cycle. (2019) 18:425–36. doi: 10.1080/15384101.2019.1568746 PMC6422466 30700227

[84] Zhang X Howell GM Guo L Collage RD Loughran PA Zuckerbraun BS. CaMKIV-dependent preservation of mTOR expression is required for autophagy during lipopolysaccharide-induced inflammation and acute kidney injury. J Immunol. (2014) 193:2405–15. doi: 10.4049/jimmunol.1302798 PMC4215705 25070845

[85] Li Y Feng G. TLR4 inhibitor alleviates sepsis-induced organ failure by inhibiting platelet mtROS production, autophagy, and GPIIb/IIIa expression. J Bioenerg Biomembr. (2022) 54:155–62. doi: 10.1007/s10863-022-09940-9 35676565

[86] Li R Zhao X Zhang S Dong W Zhang L Chen Y. RIP3 impedes transcription factor EB to suppress autophagic degradation in septic acute kidney injury. Cell Death Dis. (2021) 12:593. doi: 10.1038/s41419-021-03865-8 34103472 PMC8187512

[87] Dolma S Lessnick SL Hahn WC Stockwell BR. Identification of genotype-selective antitumor agents using synthetic lethal chemical screening in engineered human tumor cells. Cancer Cell. (2003) 3:285–96. doi: 10.1016/S1535-6108(03)00050-3 12676586

[88] Xl L Gy Z. R, G. & N, C. Ferroptosis in sepsis: The mechanism, the role and the therapeutic potential. Front Immunol. (2022) 13:956361. doi: 10.3389/fimmu.2022.956361 35990689 PMC9389368

[89] Qu M Zhang H Chen Z Sun X Zhu S Nan K. The role of ferroptosis in acute respiratory distress syndrome. Front Med (Lausanne). (2021) 8:651552. doi: 10.3389/fmed.2021.651552 34026785 PMC8137978

[90] Yu Y Yan Y Niu F Wang Y Chen X Su G. Ferroptosis: a cell death connecting oxidative stress, inflammation and cardiovascular diseases. Cell Death Discovery. (2021) 7:193. doi: 10.1038/s41420-021-00579-w 34312370 PMC8313570

[91] Wang T Fu X Chen Q Patra JK Wang D Wang Z. Arachidonic acid metabolism and kidney inflammation. Int J Mol Sci. (2019) 20:3683. doi: 10.3390/ijms20153683 31357612 PMC6695795

[92] Fang X Wang H Han D Xie E Yang X Wei J. Ferroptosis as a target for protection against cardiomyopathy. Proc Natl Acad Sci U.S.A. (2019) 116:2672–80. doi: 10.1073/pnas.1821022116 PMC6377499 30692261

[93] Gao M Monian P Quadri N Ramasamy R Jiang X. Glutaminolysis and transferrin regulate ferroptosis. Mol Cell. (2015) 59:298–308. doi: 10.1016/j.molcel.2015.06.011 26166707 PMC4506736

[94] Xie Y Hou W Song X Yu Y Huang J Sun X. Ferroptosis: process and function. Cell Death Differ. (2016) 23:369–79. doi: 10.1038/cdd.2015.158 PMC5072448 26794443

[95] Zhu H Santo A Jia Z Robert Li Y. GPx4 in bacterial infection and polymicrobial sepsis: involvement of ferroptosis and pyroptosis. React Oxyg Species (Apex). (2019) 7:154–60. doi: 10.20455/ros.2019.835 PMC6519466 31106276

[96] Ma D Jiang P Jiang Y Li H Zhang D. Effects of lipid peroxidation-mediated ferroptosis on severe acute pancreatitis-induced intestinal barrier injury and bacterial translocation. Oxid Med Cell Longevity. (2021) 2021:1–12. doi: 10.1155/2021/6644576 PMC8245223 34257815

[97] Ma R Fang L Chen L Wang X Jiang J Gao L. Ferroptotic stress promotes macrophages against intracellular bacteria. Theranostics. (2022) 12:2266–89. doi: 10.7150/thno.66663 PMC8899587 35265210

[98] Matsushita M Freigang S Schneider C Conrad M Bornkamm GW Kopf M. T cell lipid peroxidation induces ferroptosis and prevents immunity to infection. J Exp Med. (2015) 212:555–68. doi: 10.1084/jem.20140857 PMC4387287 25824823

[99] Amaral EP Costa DL Namasivayam S Riteau N Kamenyeva O Mittereder L. A major role for ferroptosis in *Mycobacterium tuberculosis* –induced cell death and tissue necrosis. . J Exp Med. (2019) 216:556–70. doi: 10.1084/jem.20181776 PMC6400546 30787033

[100] Yang Y Lin Q Zhu X Shao X Li S Li J. Activation of lipophagy is required for RAB7 to regulate ferroptosis in sepsis-induced acute kidney injury. Free Radic Biol Med. (2024) 218:120–31. doi: 10.1016/j.freeradbiomed.2024.04.213 38583680

[101] Feng W Zhu N Xia Y Huang Z Hu J Guo Z. Melanin-like nanoparticles alleviate ischemia-reperfusion injury in the kidney by scavenging reactive oxygen species and inhibiting ferroptosis. iScience. (2024) 27:109504. doi: 10.1016/j.isci.2024.109504 38632989 PMC11022057

[102] Luo R-R Yang J Sun YL Zhou BY Zhou SX Zhang GX. Dexmedetomidine attenuates ferroptosis by Keap1-Nrf2/HO-1 pathway in LPS-induced acute kidney injury. Naunyn Schmiedebergs Arch Pharmacol. (2024) 397:7785–96. doi: 10.1007/s00210-024-03125-4 38722344

[103] Bergmann O Bhardwaj RD Bernard S Zdunek S Barnabé-Heider F Walsh S. Evidence for cardiomyocyte renewal in humans. Science. (2009) 324:98–102. doi: 10.1126/science.1164680 19342590 PMC2991140

[104] Dixon SJ Lemberg KM Lamprecht MR Skouta R Zaitsev EM Gleason CE. Ferroptosis: an iron-dependent form of nonapoptotic cell death. Cell. (2012) 149:1060–72. doi: 10.1016/j.cell.2012.03.042 PMC3367386 22632970

[105] Xie Y Kang R Klionsky DJ Tang D. GPX4 in cell death, autophagy, and disease. Autophagy. (2023) 19:2621–38. doi: 10.1080/15548627.2023.2218764 PMC10472888 37272058

[106] Flohé L Toppo S Orian L. The glutathione peroxidase family: Discoveries and mechanism. Free Radic Biol Med. (2022) 187:113–22. doi: 10.1016/j.freeradbiomed.2022.05.003 35580774

[107] Li F-J Long HZ Zhou ZW Luo HY Xu SG Gao LC. System Xc -/GSH/GPX4 axis: An important antioxidant system for the ferroptosis in drug-resistant solid tumor therapy. Front Pharmacol. (2022) 13:910292. doi: 10.3389/fphar.2022.910292 36105219 PMC9465090

[108] Yu H Guo P Xie X Wang Y Chen G. Ferroptosis, a new form of cell death, and its relationships with tumourous diseases. J Cell Mol Med. (2017) 21:648–57. doi: 10.1111/jcmm.2017.21.issue-4 PMC5345622 27860262

[109] Bogdan AR Miyazawa M Hashimoto K Tsuji Y. Regulators of iron homeostasis: new players in metabolism, cell death, and disease. Trends Biochem Sci. (2016) 41:274–86. doi: 10.1016/j.tibs.2015.11.012 PMC4783254 26725301

[110] Li N Wang W Zhou H Wu Q Duan M Liu C. Ferritinophagy-mediated ferroptosis is involved in sepsis-induced cardiac injury. Free Radic Biol Med. (2020) 160:303–18. doi: 10.1016/j.freeradbiomed.2020.08.009 32846217

[111] Qiongyue Z. Post-treatment with irisin attenuates acute kidney injury in sepsis mice through anti-ferroptosis via the SIRT1/nrf2 pathway. Front Pharmacol. (2022) 13:857067. doi: 10.3389/fphar.2022.857067 35370723 PMC8970707

[112] Zhou Z Zhang H. CHAC1 exacerbates LPS-induced ferroptosis and apoptosis in HK-2 cells by promoting oxidative stress. Allergol Immunopathol (Madr). (2023) 51:99–110. doi: 10.15586/aei.v51i2.760 36916093

[113] Koppula P Zhuang L Gan B. Cystine transporter SLC7A11/xCT in cancer: ferroptosis, nutrient dependency, and cancer therapy. Protein Cell. (2021) 12:599–620. doi: 10.1007/s13238-020-00789-5 33000412 PMC8310547

[114] Jiang X Stockwell BR Conrad M. Ferroptosis: mechanisms, biology and role in disease. Nat Rev Mol Cell Biol. (2021) 22:266–82. doi: 10.1038/s41580-020-00324-8 PMC8142022 33495651

[115] Zhang Y Zeng Y Huang M Cao G Lin L Wang X. Andrographolide attenuates sepsis-induced acute kidney injury by inhibiting ferroptosis through the Nrf2/FSP1 pathway. Free Radic Res. (2024) 58:156–69. doi: 10.1080/10715762.2024.2330413 38478853

[116] Gardiner EE Andrews RK. Neutrophil extracellular traps (NETs) and infection-related vascular dysfunction. Blood Rev. (2012) 26:255–9. doi: 10.1016/j.blre.2012.09.001 23021640

[117] Erpenbeck L Schön MP. Neutrophil extracellular traps: protagonists of cancer progression? Oncogene. (2017) 36:2483–90. doi: 10.1038/onc.2016.406 27941879

[118] Douda DN Yip L Khan MA Grasemann H Palaniyar N. Akt is essential to induce NADPH-dependent NETosis and to switch the neutrophil death to apoptosis. Blood. (2014) 123:597–600. doi: 10.1182/blood-2013-09-526707 24458280

[119] Ravindran M Khan MA Palaniyar N. Neutrophil extracellular trap formation: physiology, pathology, and pharmacology. Biomolecules. (2019) 9:365. doi: 10.3390/biom9080365 31416173 PMC6722781

[120] Azzouz D Khan MA Sweezey N Palaniyar N. Two-in-one: UV radiation simultaneously induces apoptosis and NETosis. Cell Death Discovery. (2018) 4:51. doi: 10.1038/s41420-018-0048-3 PMC5919968 29736268

[121] Rohrbach AS Slade DJ Thompson PR Mowen KA. Activation of PAD4 in NET formation. Front Immunol. (2012) 3:360. doi: 10.3389/fimmu.2012.00360 23264775 PMC3525017

[122] Douda DN Khan MA Grasemann H Palaniyar N. SK3 channel and mitochondrial ROS mediate NADPH oxidase-independent NETosis induced by calcium influx. Proc Natl Acad Sci U.S.A. (2015) 112:2817–22. doi: 10.1073/pnas.1414055112 PMC4352781 25730848

[123] Azzouz D Palaniyar N. ApoNETosis: discovery of a novel form of neutrophil death with concomitant apoptosis and NETosis. Cell Death Dis. (2018) 9:839. doi: 10.1038/s41419-018-0846-9 30082793 PMC6079026

[124] Metzler KD Goosmann C Lubojemska A Zychlinsky A. amp]]amp; Papayannopoulos, V. A myeloperoxidase-containing complex regulates neutrophil elastase release and actin dynamics during NETosis. Cell Rep. (2014) 8:883–96. doi: 10.1016/j.celrep.2014.06.044 PMC4471680 25066128

[125] Khan MA Palaniyar N. Transcriptional firing helps to drive NETosis. Sci Rep. (2017) 7:41749. doi: 10.1038/srep41749 28176807 PMC5296899

[126] Papayannopoulos V. Neutrophil extracellular traps in immunity and disease. Nat Rev Immunol. (2018) 18:134–47. doi: 10.1038/nri.2017.105 28990587

[127] Allam R Scherbaum CR Darisipudi MN Mulay SR Hägele H Lichtnekert J. Histones from dying renal cells aggravate kidney injury via TLR2 and TLR4. J Am Soc Nephrol. (2012) 23:1375–88. doi: 10.1681/ASN.2011111077 PMC3402284 22677551

[128] Ekaney ML Otto GP Sossdorf M Sponholz C Boehringer M Loesche W. Impact of plasma histones in human sepsis and their contribution to cellular injury and inflammation. Crit Care. (2014) 18:543. doi: 10.1186/s13054-014-0543-8 25260379 PMC4201918

[129] Kumar SVR Kulkarni OP Mulay SR Darisipudi MN Romoli S Thomasova D. Neutrophil extracellular trap-related extracellular histones cause vascular necrosis in severe GN. J Am Soc Nephrol. (2015) 26:2399–413. doi: 10.1681/ASN.2014070673 PMC4587690 25644111

[130] Nakazawa D Kumar SV Marschner J Desai J Holderied A Rath L. Histones and neutrophil extracellular traps enhance tubular necrosis and remote organ injury in ischemic AKI. J Am Soc Nephrol. (2017) 28:1753–68. doi: 10.1681/ASN.2016080925 PMC5461800 28073931

[131] Allam R Kumar SVR Darisipudi MN Anders H-J. Extracellular histones in tissue injury and inflammation. J Mol Med (Berl). (2014) 92:465–72. doi: 10.1007/s00109-014-1148-z 24706102

[132] Everts B Amiel E Huang SCC Smith AM Chang CH Lam WY. TLR-driven early glycolytic reprogramming via the kinases TBK1-IKKε supports the anabolic demands of dendritic cell activation. Nat Immunol. (2014) 15:323–32. doi: 10.1038/ni.2833 PMC4358322 24562310

[133] Soto-Heredero G Gómez De Las Heras MM Gabandé-Rodríguez E Oller J Mittelbrunn M. Glycolysis – a key player in the inflammatory response. FEBS J. (2020) 287:3350–69. doi: 10.1111/febs.15327 PMC7496292 32255251

[134] Lan R Geng H Singha PK Saikumar P Bottinger EP Weinberg JM. Mitochondrial Pathology and Glycolytic Shift during Proximal Tubule Atrophy after Ischemic AKI. JASN. (2016) 27:3356–67. doi: 10.1681/ASN.2015020177 PMC5084876 27000065

[135] Lunt SY Vander Heiden MG. Aerobic glycolysis: meeting the metabolic requirements of cell proliferation. Annu Rev Cell Dev Biol. (2011) 27:441–64. doi: 10.1146/annurev-cellbio-092910-154237 21985671

[136] Macintyre AN Gerriets VA Nichols AG Michalek RD Rudolph MC Deoliveira D. The glucose transporter glut1 is selectively essential for CD4 T cell activation and effector function. Cell Metab. (2014) 20:61–72. doi: 10.1016/j.cmet.2014.05.004 24930970 PMC4079750

[137] Freemerman AJ Johnson AR Sacks GN Milner JJ Kirk EL Troester MA. Metabolic reprogramming of macrophages. J Biol Chem. (2014) 289:7884–96. doi: 10.1074/jbc.M113.522037 PMC3953299 24492615

[138] Michalek RD Gerriets VA Jacobs SR Macintyre AN MacIver NJ Mason EF. Cutting edge: distinct glycolytic and lipid oxidative metabolic programs are essential for effector and regulatory CD4+ T cell subsets. J Immunol. (2011) 186:3299–303. doi: 10.4049/jimmunol.1003613 PMC3198034 21317389

[139] Gauthier T Chen W. Modulation of macrophage immunometabolism: A new approach to fight infections. Front Immunol. (2022) 13:780839. doi: 10.3389/fimmu.2022.780839 35154105 PMC8825490

[140] Pålsson-McDermott EM O’Neill LAJ. Targeting immunometabolism as an anti-inflammatory strategy. Cell Res. (2020) 30:300–14. doi: 10.1038/s41422-020-0291-z PMC7118080 32132672

[141] Kelly B Tannahill GM Murphy MP O’Neill LAJ. Metformin inhibits the production of reactive oxygen species from NADH: ubiquinone oxidoreductase to limit induction of interleukin-1β (IL-1β) and boosts interleukin-10 (IL-10) in lipopolysaccharide (LPS)-activated macrophages. J Biol Chem. (2015) 290:20348–59. doi: 10.1074/jbc.M115.662114 PMC4536441 26152715

[142] Bauernfeind F Bartok E Rieger A Franchi L Núñez G Hornung V. Cutting edge: reactive oxygen species inhibitors block priming, but not activation, of the NLRP3 inflammasome. J Immunol. (2011) 187:613–7. doi: 10.4049/jimmunol.1100613 PMC3131480 21677136

[143] Cheng S-C Quintin J Cramer RA Shepardson KM Saeed S Kumar V. mTOR- and HIF-1α–mediated aerobic glycolysis as metabolic basis for trained immunity. Science. (2014) 345:1250684. doi: 10.1126/science.1250684 25258083 PMC4226238

[144] Kasprzak A. Insulin-like growth factor 1 (IGF-1) signaling in glucose metabolism in colorectal cancer. IJMS. (2021) 22:6434. doi: 10.3390/ijms22126434 34208601 PMC8234711

[145] Torp M-K Yang K Ranheim T Husø Lauritzen K Alfsnes K Vinge LE. Mammalian target of rapamycin (mTOR) and the proteasome attenuates IL-1β Expression in primary mouse cardiac fibroblasts. Front Immunol. (2019) 10:1285. doi: 10.3389/fimmu.2019.01285 31244838 PMC6563870

[146] Delgoffe GM Kole TP Zheng Y Zarek PE Matthews KL Xiao B. The mTOR kinase differentially regulates effector and regulatory T cell lineage commitment. Immunity. (2009) 30:832–44. doi: 10.1016/j.immuni.2009.04.014 PMC2768135 19538929

[147] Sukumar M Liu J Ji Y Subramanian M Crompton JG Yu Z. Inhibiting glycolytic metabolism enhances CD8+ T cell memory and antitumor function. J Clin Invest.(2013) 123:4479–88. doi: 10.1172/JCI69589 PMC3784544 24091329

[148] Zhang Q Hu Y Zhang J Deng C. iTRAQ−based proteomic analysis of endotoxin tolerance induced by lipopolysaccharide. Mol Med Rep. (2019) 20:584–92. doi: 10.3892/mmr.2019.10264 PMC6580005 31115557

[149] Morris M Li L. Molecular mechanisms and pathological consequences of endotoxin tolerance and priming. Arch Immunol Ther Exp. (2012) 60:13–8. doi: 10.1007/s00005-011-0155-9 22143158

[150] Liu TF Vachharajani VT Yoza BK McCall CE. NAD+-dependent sirtuin 1 and 6 proteins coordinate a switch from glucose to fatty acid oxidation during the acute inflammatory response. J Biol Chem. (2012) 287:25758–69. doi: 10.1074/jbc.M112.362343 PMC3406663 22700961

[151] Manoharan I Prasad PD Thangaraju M Manicassamy S. Lactate-dependent regulation of immune responses by dendritic cells and macrophages. Front Immunol. (2021) 12:691134. doi: 10.3389/fimmu.2021.691134 34394085 PMC8358770

[152] Brooks GA. The tortuous path of lactate shuttle discovery: From cinders and boards to the lab and ICU. J Sport Health Sci. (2020) 9:446–60. doi: 10.1016/j.jshs.2020.02.006 PMC7498672 32444344

[153] Chen P Zuo H Xiong H Kolar MJ Chu Q Saghatelian A. Gpr132 sensing of lactate mediates tumor–macrophage interplay to promote breast cancer metastasis. Proc Natl Acad Sci USA. (2017) 114:580–5. doi: 10.1073/pnas.1614035114 PMC5255630 28049847

[154] Errea A Cayet D Marchetti P Tang C Kluza J Offermanns S. Lactate inhibits the pro-inflammatory response and metabolic reprogramming in murine macrophages in a GPR81-independent manner. PloS One. (2016) 11:e0163694. doi: 10.1371/journal.pone.0163694 27846210 PMC5112849

[155] Hoque R Farooq A Ghani A Gorelick F Mehal WZ. Lactate reduces liver and pancreatic injury in toll-like receptor– and inflammasome-mediated inflammation via GPR81-mediated suppression of innate immunity. Gastroenterology. (2014) 146:1763–74. doi: 10.1053/j.gastro.2014.03.014 PMC4104305 24657625

[156] Dietl K Renner K Dettmer K Timischl B Eberhart K Dorn C. Lactic acid and acidification inhibit TNF secretion and glycolysis of human monocytes. J Immunol. (2010) 184:1200–9. doi: 10.4049/jimmunol.0902584 20026743

[157] Yang K Xu J Fan M Tu F Wang X Ha T. Lactate suppresses macrophage pro-inflammatory response to LPS stimulation by inhibition of YAP and NF-κB activation via GPR81-mediated signaling. Front Immunol. (2020) 11:587913. doi: 10.3389/fimmu.2020.587913 33123172 PMC7573489

[158] Haas R Smith J Rocher-Ros V Nadkarni S Montero-Melendez T D’Acquisto F. Lactate regulates metabolic and pro-inflammatory circuits in control of T cell migration and effector functions. PloS Biol. (2015) 13:e1002202. doi: 10.1371/journal.pbio.1002202 26181372 PMC4504715

[159] Khaliq W Großmann P Neugebauer S Kleyman A Domizi R Calcinaro S. Lipid metabolic signatures deviate in sepsis survivors compared to non-survivors. Comput Struct Biotechnol J. (2020) 18:3678–91. doi: 10.1016/j.csbj.2020.11.009 PMC7711192 33304464

[160] Iwaki T Bennion BG Stenson EK Lynn JC Otinga C Djukovic D. PPAR *α* contributes to protection against metabolic and inflammatory derangements associated with acute kidney injury in experimental sepsis. Physiol Rep. (2019) 7:e14078. doi: 10.14814/phy2.14078 31102342 PMC6525329

[161] Bhargava P Schnellmann RG. Mitochondrial energetics in the kidney. Nat Rev Nephrol. (2017) 13:629–46. doi: 10.1038/nrneph.2017.107 PMC5965678 28804120

[162] Harzandi A Lee S Bidkhori G Saha S Hendry BM Mardinoglu A. Acute kidney injury leading to CKD is associated with a persistence of metabolic dysfunction and hypertriglyceridemia. iScience. (2021) 24:102046. doi: 10.1016/j.isci.2021.102046 33554059 PMC7843454

[163] Han SH Wu yan Nam M BY Park JT Yoo TH Kang SW. PGC-1α Protects from notch-induced kidney fibrosis development. JASN. (2017) 28:3312–22. doi: 10.1681/ASN.2017020130 PMC5661291 28751525

[164] Yang X Okamura DM Lu X Chen Y Moorhead J Varghese Z. CD36 in chronic kidney disease: novel insights and therapeutic opportunities. Nat Rev Nephrol. (2017) 13:769–81. doi: 10.1038/nrneph.2017.126 28919632

[165] Houten SM Violante S Ventura FV Wanders RJA. The biochemistry and physiology of mitochondrial fatty acid β-oxidation and its genetic disorders. Annu Rev Physiol. (2016) 78:23–44. doi: 10.1146/annurev-physiol-021115-105045 26474213

[166] Gao Z Chen X. Fatty acid β-oxidation in kidney diseases: perspectives on pathophysiological mechanisms and therapeutic opportunities. Front Pharmacol. (2022) 13:805281. doi: 10.3389/fphar.2022.805281 35517820 PMC9065343

[167] Kalakeche R Hato T Rhodes G Dunn KW El-Achkar TM Plotkin Z. Endotoxin uptake by S1 proximal tubular segment causes oxidative stress in the downstream S2 segment. J Am Soc Nephrol. (2011) 22:1505–16. doi: 10.1681/ASN.2011020203 PMC3148705 21784899

[168] Dellepiane S Marengo M Cantaluppi V. Detrimental cross-talk between sepsis and acute kidney injury: new pathogenic mechanisms, early biomarkers and targeted therapies. Crit Care. (2016) 20:61. doi: 10.1186/s13054-016-1219-3 26976392 PMC4792098

[169] Li Y Nourbakhsh N Pham H Tham R Zuckerman JE Singh P. Evolution of altered tubular metabolism and mitochondrial function in sepsis-associated acute kidney injury. Am J Physiology-Renal Physiol. (2020) 319:F229–44. doi: 10.1152/ajprenal.00390.2019 PMC7473900 32538150

[170] Waltz P Carchman E Gomez H Zuckerbraun B. Sepsis results in an altered renal metabolic and osmolyte profile. J Surg Res. (2016) 202:8–12. doi: 10.1016/j.jss.2015.12.011 27083942

[171] Liu J Zhou G Wang X Liu D. Metabolic reprogramming consequences of sepsis: adaptations and contradictions. Cell Mol Life Sci. (2022) 79:456. doi: 10.1007/s00018-022-04490-0 35904600 PMC9336160

[172] Jang H-S Noh MR Kim J Padanilam BJ. Defective mitochondrial fatty acid oxidation and lipotoxicity in kidney diseases. Front Med. (2020) 7:65. doi: 10.3389/fmed.2020.00065 PMC7080698 32226789

[173] Gai Z Wang T Visentin M Kullak-Ublick GA Fu X Wang Z. Lipid accumulation and chronic kidney disease. Nutrients. (2019) 11:722. doi: 10.3390/nu11040722 30925738 PMC6520701

[174] Xiong W Xiong Z Song A Lei C Ye C Zhang C. Relieving lipid accumulation through UCP1 suppresses the progression of acute kidney injury by promoting the AMPK/ULK1/autophagy pathway. Theranostics. (2021) 11:4637–54. doi: 10.7150/thno.56082 PMC7978316 33754018

[175] Toro J Manrique-Caballero CL Gómez H. Metabolic reprogramming and host tolerance: A novel concept to understand sepsis-associated AKI. JCM. (2021) 10:4184. doi: 10.3390/jcm10184184 34575294 PMC8471000

[176] Wang Y Cui C Zhao W Tian X Liu P Wei L. WIP1-mediated regulation of p38 MAPK signaling attenuates pyroptosis in sepsis-associated acute kidney injury. Immunobiology. (2024) 229:152832. doi: 10.1016/j.imbio.2024.152832 38943814

[177] Guo J Yuan Z Wang R. Zn2+ improves sepsis-induced acute kidney injury by upregulating SIRT7-mediated Parkin acetylation. Am J Physiol Renal Physiol. (2024) 327:F184–97. doi: 10.1152/ajprenal.00337.2023 38779758

[178] Fang S Sun R Su H Zhai K Xiang Y Gao Y. Chlorogenic acid alleviates acute kidney injury in septic mice by inhibiting NLRP3 inflammasomes and the caspase-1 canonical pyroptosis pathway. Nan Fang Yi Ke Da Xue Xue Bao. (2024) 44:317–23. doi: 10.12122/j.issn.1673-4254.2024.02.14 PMC10954528 38501417

[179] Sun M Wang F Li H Li M Wang Y Wang C. Maresin-1 attenuates sepsis-associated acute kidney injury via suppressing inflammation, endoplasmic reticulum stress and pyroptosis by activating the AMPK/SIRT3 pathway. J Inflammation Res. (2024) 17:1349–64. doi: 10.2147/JIR.S442729 PMC10908291 38434585

[180] Songür HS Kaya SA Altınışık YC Abanoz R Özçelebi E Özmen F. Alamandine treatment prevents LPS-induced acute renal and systemic dysfunction with multi-organ injury in rats via inhibiting iNOS expression. Eur J Pharmacol. (2023) 960:176160. doi: 10.1016/j.ejphar.2023.176160 37923157

[181] Yang Y Xu J Tu J Sun Y Zhang C Qiu Z. Polygonum cuspidatum Sieb. et Zucc. Extracts improve sepsis-associated acute kidney injury by inhibiting NF-κB-mediated inflammation and pyroptosis. J Ethnopharmacol. (2024) 319:117101. doi: 10.1016/j.jep.2023.117101 37657770

[182] Liu Y Fang Q Ming T Zuo J Jing G Song X. Knockout of Erbin promotes pyroptosis via regulating NLRP3/caspase-1/Gasdermin D pathway in sepsis-induced acute kidney injury. Clin Exp Nephrol. (2023) 27:781–90. doi: 10.1007/s10157-023-02364-8 37310569

[183] Chen S-Y Li YP You YP Zhang HR Shi ZJ Liang QQ. Theaflavin mitigates acute gouty peritonitis and septic organ injury in mice by suppressing NLRP3 inflammasome assembly. Acta Pharmacol Sin. (2023) 44:2019–36. doi: 10.1038/s41401-023-01105-7 PMC10545837 37221235

[184] Francis MR El-Sheakh AR Suddek GM. Saroglitazar, a dual PPAR-α/γ agonist, alleviates LPS-induced hepatic and renal injury in rats. Int Immunopharmacol. (2023) 115:109688. doi: 10.1016/j.intimp.2023.109688 36681027

[185] Zhou J Zhang F Lin H Quan M Yang Y Lv Y. The protein kinase R inhibitor C16 alleviates sepsis-induced acute kidney injury through modulation of the NF-κB and NLR family pyrin domain-containing 3 (NLPR3) pyroptosis signal pathways. Med Sci Monit. (2020) 26:e926254. doi: 10.12659/MSM.926254 33017381 PMC7545781

[186] Liu R Wang SC Li M Ma XH Jia XN Bu Y. An inhibitor of DRP1 (Mdivi-1) alleviates LPS-induced septic AKI by inhibiting NLRP3 inflammasome activation. BioMed Res Int. (2020) 2020:2398420. doi: 10.1155/2020/2398420 32733934 PMC7369665

[187] Guo L-P Liu SX Yang Q Liu HY Xu LL Hao YH. Effect of thymoquinone on acute kidney injury induced by sepsis in BALB/c mice. BioMed Res Int. (2020) 2020:1594726. doi: 10.1155/2020/1594726 32626733 PMC7315249

[188] Lei X Wang J Zhang F Tang X He F Cheng S. Micheliolide ameliorates lipopolysaccharide-induced acute kidney injury through suppression of NLRP3 activation by promoting mitophagy via Nrf2/PINK1/Parkin axis. Int Immunopharmacol. (2024) 138:112527. doi: 10.1016/j.intimp.2024.112527 38950457

[189] Ling Z-Y Lv QZ Li J Lu RY Chen LL Xu WH. Protective effect of a novel RIPK1 inhibitor, compound 4-155, in systemic inflammatory response syndrome and sepsis. Inflammation. (2023) 46:1796–809. doi: 10.1007/s10753-023-01842-1 37227549

[190] Li X Zeng Q Yao R Zhang L Kong Y Shen B. Rapamycin mitigates organ damage by autophagy-mediated NLRP3 inflammasome inactivation in sepsis. Histol Histopathol. (2024) 39:1167–77. doi: 10.14670/HH-18-706 38288570

[191] Yang N Yan N Bai Z Du S Zhang J Zhang L. FTO attenuates LPS-induced acute kidney injury by inhibiting autophagy via regulating SNHG14/miR-373-3p/ATG7 axis. Int Immunopharmacol. (2024) 128:111483. doi: 10.1016/j.intimp.2023.111483 38215656

[192] Zheng Y Cai JJ Yang X Shao ZQ Liu JQ Yang XH. Alcohol dehydrogenase 1 is a tubular mitophagy-dependent apoptosis inhibitor against septic acute kidney injury. Exp Cell Res. (2023) 433:113804. doi: 10.1016/j.yexcr.2023.113804 37806378

[193] Zhang W Chen H Xu Z Zhang X Tan X He N. Liensinine pretreatment reduces inflammation, oxidative stress, apoptosis, and autophagy to alleviate sepsis acute kidney injury. Int Immunopharmacol. (2023) 122:110563. doi: 10.1016/j.intimp.2023.110563 37392573

[194] Li T Ji X Liu J Guo X Pang R Zhuang H. Ulinastatin improves renal microcirculation by protecting endothelial cells and inhibiting autophagy in a septic rat model. Kidney Blood Press Res. (2022) 47:256–69. doi: 10.1159/000521648 35016182

[195] Chen ZD Hu BC Shao XP Hong J Zheng Y Zhang R. Ascorbate uptake enables tubular mitophagy to prevent septic AKI by PINK1-PARK2 axis. Biochem Biophys Res Commun. (2021) 554:158–65. doi: 10.1016/j.bbrc.2021.03.103 33798942

[196] Liu J-X Yang C Liu ZJ Su HY Zhang WH Pan Q. Protection of procyanidin B2 on mitochondrial dynamics in sepsis associated acute kidney injury via promoting Nrf2 nuclear translocation. Aging (Albany NY). (2020) 12:15638–55. doi: 10.18632/aging.103726 PMC7467384 32805725

[197] Yang T Feng X Zhao Y Zhang H Cui H Wei M. Dexmedetomidine enhances autophagy via α2-AR/AMPK/mTOR pathway to inhibit the activation of NLRP3 inflammasome and subsequently alleviates lipopolysaccharide-induced acute kidney injury. Front Pharmacol. (2020) 11:790. doi: 10.3389/fphar.2020.00790 32670056 PMC7326938

[198] Zhou P Zhao C Chen Y Liu X Wu C Hu Z. Klotho activation of Nrf2 inhibits the ferroptosis signaling pathway to ameliorate sepsis-associated acute kidney injury. Transl Androl Urol. (2023) 12:1871–84. doi: 10.21037/tau-23-573 PMC10772648 38196698

[199] Zan H Liu J Yang M Zhao H Gao C Dai Y. Melittin alleviates sepsis-induced acute kidney injury by promoting GPX4 expression to inhibit ferroptosis. Redox Rep. (2024) 29:2290864. doi: 10.1080/13510002.2023.2290864 38149613 PMC10763831

[200] Li J Liu Y Bai J Liu T Qin X Hu T. Dexmedetomidine alleviates renal tubular ferroptosis in sepsis-associated AKI by KEAP1 regulating the degradation of GPX4. Eur J Pharmacol. (2023) 961:176194. doi: 10.1016/j.ejphar.2023.176194 38000722

[201] Zhang L Rao J Liu X Wang X Wang C Fu S. Attenuation of sepsis-induced acute kidney injury by exogenous H2S via inhibition of ferroptosis. Molecules. (2023) 28:4770. doi: 10.3390/molecules28124770 37375325 PMC10305203

[202] Qiu W An S Wang T Li J Yu B Zeng Z. Melatonin suppresses ferroptosis via activation of the Nrf2/HO-1 signaling pathway in the mouse model of sepsis-induced acute kidney injury. Int Immunopharmacol. (2022) 112:109162. doi: 10.1016/j.intimp.2022.109162 36067654

[203] Guo J Wang R Min F. Ginsenoside Rg1 ameliorates sepsis-induced acute kidney injury by inhibiting ferroptosis in renal tubular epithelial cells. J Leukoc Biol. (2022) 112:1065–77. doi: 10.1002/JLB.1A0422-211R 35774015

[204] Xiao J Yang Q Zhang Y Xu H Ye Y Li L. Maresin conjugates in tissue regeneration-1 suppresses ferroptosis in septic acute kidney injury. Cell Biosci. (2021) 11:221. doi: 10.1186/s13578-021-00734-x 34961563 PMC8711186

[205] Tan C Gu J Li T Chen H Liu K Liu M. Inhibition of aerobic glycolysis alleviates sepsis−induced acute kidney injury by promoting lactate/Sirtuin 3/AMPK−regulated autophagy. Int J Mol Med. (2021) 47:19. doi: 10.3892/ijmm.2021.4852 33448325 PMC7849980

[206] Ji R Chen W Wang Y Gong F Huang S Zhong M. The warburg effect promotes mitochondrial injury regulated by uncoupling protein-2 in septic acute kidney injury. Shock. (2021) 55:640–8. doi: 10.1097/SHK.0000000000001576 32496419

[207] Yang M Lopez LN Brewer M Delgado R Menshikh A Clouthier K. Inhibition of Retinoic Acid Signaling in Proximal Tubular Epithelial cells Protects against Acute Kidney Injury by Enhancing Kim-1-dependent Efferocytosis. bioRxiv. (2023) 2023:6.15.545113. doi: 10.1101/2023.06.15.545113 PMC10619506 37698919

[208] Dai Q Vitamin D-VDR. (vitamin D receptor) alleviates glucose metabolism reprogramming in lipopolysaccharide-induced acute kidney injury. Front Physiol. (2023) 14:1083643. doi: 10.3389/fphys.2023.1083643 36909229 PMC9998528

[209] Jin K Ma Y Manrique-Caballero CL Li H Emlet DR Li S. Activation of AMP-activated protein kinase during sepsis/inflammation improves survival by preserving cellular metabolic fitness. FASEB J. (2020) 34:7036–57. doi: 10.1096/fj.201901900R PMC11956121 32246808

[210] Wu J Rong S Zhou J Yuan,W. The role and mechanism of PKM2 in the development of LPS-induced acute kidney injury. Histol Histopathol. (2021) 36:845–52. doi: 10.14670/HH-18-343 33978226

[211] Luo P Zhang Q Zhong TY Chen JY Zhang JZ Tian Y. Celastrol mitigates inflammation in sepsis by inhibiting the PKM2-dependent Warburg effect. Military Med Res. (2022) 9:22. doi: 10.1186/s40779-022-00381-4 PMC9121578 35596191

